# Resistance to allosteric SHP2 inhibition in FGFR-driven cancers through rapid feedback activation of FGFR

**DOI:** 10.18632/oncotarget.27435

**Published:** 2020-01-21

**Authors:** Hengyu Lu, Chen Liu, Hung Huynh, Thi Bich Uyen Le, Matthew J. LaMarche, Morvarid Mohseni, Jeffrey A. Engelman, Peter S. Hammerman, Giordano Caponigro, Huai-Xiang Hao

**Affiliations:** ^1^ Novartis Institutes for Biomedical Research, Oncology Disease Area, Cambridge, Massachusetts, USA; ^2^ Laboratory of Molecular Endocrinology, Division of Molecular and Cellular Research, National Cancer Centre, Singapore; ^3^ Novartis Institutes for Biomedical Research, Global Discovery Chemistry, Cambridge, Massachusetts, USA

**Keywords:** SHP2, FGFR, resistance, feedback activation

## Abstract

SHP2 mediates RAS activation downstream of multiple receptor tyrosine kinases (RTKs) and cancer cell lines dependent on RTKs are in general dependent on SHP2. Profiling of the allosteric SHP2 inhibitor SHP099 across cancer cell lines harboring various RTK dependencies reveals that FGFR-dependent cells are often insensitive to SHP099 when compared to EGFR-dependent cells. We find that FGFR-driven cells depend on SHP2 but exhibit resistance to SHP2 inhibitors *in vitro* and *in vivo*. Treatment of such models with SHP2 inhibitors results in an initial decrease in phosphorylated ERK1/2 (p-ERK) levels, however p-ERK levels rapidly rebound within two hours. This p-ERK rebound is blocked by FGFR inhibitors or high doses of SHP2 inhibitors. Mechanistically, compared with EGFR-driven cells, FGFR-driven cells tend to express high levels of RTK negative regulators such as the SPRY family proteins, which are rapidly downregulated upon ERK inhibition. Moreover, over-expression of SPRY4 in FGFR-driven cells prevents MAPK pathway reactivation and sensitizes them to SHP2 inhibitors. We also identified two novel combination approaches to enhance the efficacy of SHP2 inhibitors, either with a distinct site 2 allosteric SHP2 inhibitor or with a RAS-SOS1 interaction inhibitor. Our findings suggest the rapid FGFR feedback activation following initial pathway inhibition by SHP2 inhibitors may promote the open conformation of SHP2 and lead to resistance to SHP2 inhibitors. These findings may assist to refine patient selection and predict resistance mechanisms in the clinical development of SHP2 inhibitors and to suggest strategies for discovering SHP2 inhibitors that are more effective against upstream feedback activation.

## INTRODUCTION

The non-receptor protein tyrosine phosphatase SHP2, encoded by the *PTPN11* gene, is ubiquitously expressed and plays an important role in multiple signaling pathways regulating cell survival, motility and proliferation. One of the most characterized functions of SHP2 is the activation of the RAS-mitogen-activated protein kinase (MAPK) signaling pathway downstream of various receptor tyrosine kinases (RTKs) [1–5]. SHP2 consists of two Src homology 2 (SH2) domains (N-terminal SH2 and C-terminal SH2), a catalytic phosphatase (PTP) domain, and a C-terminal tail with two tyrosine phosphorylation sites (Y542 and Y580) [6–8]. In its inactive state, SHP2 adopts a closed autoinhibitory conformation, where the catalytic site is hindered by the N-terminal SH2 domain. When phosphotyrosyl peptides bind to its SH2 domains, SHP2 adopts an open and active conformation thereby exposing its catalytic site. SHP2 binding sites are found in RTKs and their adaptor proteins such as GAB1, GRB2, and others, which form a complex in response to RTK activation and promote RAS activation by recruiting its guanine exchange factors (GEFs) such as SOS1 to the membrane. SHP2 can be phosphorylated at Y542 and Y580 as a result of RTK activation, which may promote SHP2 activity [9]. Given the importance of RAS-MAPK signaling downstream of RTK, it is not surprising that RTK-dependent cancer cells are often sensitive to SHP2 depletion [10]. Allosteric SHP2 inhibitors such as SHP099 and SHP394 stabilize the closed auto-inhibited state [11, 12], which effectively inhibit the RAS-MAPK signaling pathway in cancer cells driven by epidermal growth factor receptors (EGFR) and other RTKs and their growth *in vitro* and *in vivo* [10, 12]. SHP2 inhibitors provide a unique opportunity to target various RTK-dependent cancers and RTK-mediated resistance mechanism to targeted therapies.

A recent study reported that fibroblast growth factor receptors (FGFRs) may activate RAS in a SHP2-independent manner in BRAF mutant colon and thyroid cancer cells in the setting of pathway feedback activation following treatment with BRAF inhibitors such as vemurafenib [13]. The conclusion was based on the ineffectiveness of up to 10 μM SHP099 to prevent the FGFR-driven reactivation of ERK and the lack of detectable basal and vemurafenib-induced SHP2 phosphorylation in three BRAF mutant cell lines [13]. This observation contrasted with published data describing a prominent role for SHP2 in FGFR-driven MAPK signaling [14, 15]. The FGFR family contains four members (FGFR1-4), which interact with a diverse set of at least 22 ligands (fibroblast growth factors, FGFs) collectively forming a complicated set of FGF-FGFR pairs that may differ in how they transduce downstream signaling such as recruiting different adaptor complexes [16, 17]. Unlike other RTKs, FGFRs require a unique adaptor molecule FGFR substrate 2 (FRS2), which has been shown to bind to SHP2 and other adaptors such as GRB2, for activating downstream signaling pathways [14, 15].

To investigate the sensitivity of various FGFR-dependent cell lines to allosteric SHP2 inhibition, we examined the correlation between sensitivity to SHP099 and sensitivity to a variety of RTK inhibitors in a high-throughput compound profiling of cancer cell lines as previously described [18, 19]. We found and confirmed that MAPK-dependent cells driven by FGFRs were resistant to SHP2 inhibitors compared with those driven by EGFR. Intriguingly, those FGFR-driven cells are genetically dependent on SHP2. In this study, we found the rapid FGFR-mediated feedback activation of ERK within two hours of SHP2 inhibition may explain the disconnect between genetic dependency and pharmacological resistance. We further demonstrated that higher baseline expression and more rapid downregulation of the SPRY proteins, negative regulators of FGFR and other RTKs, were at least partially responsible for the rapid feedback activation of FGFRs compared with EGFR-dependent cells.

## RESULTS

### FGFR-driven MAPK-dependent cells are resistant to allosteric SHP2 inhibition

**Figure 1:**
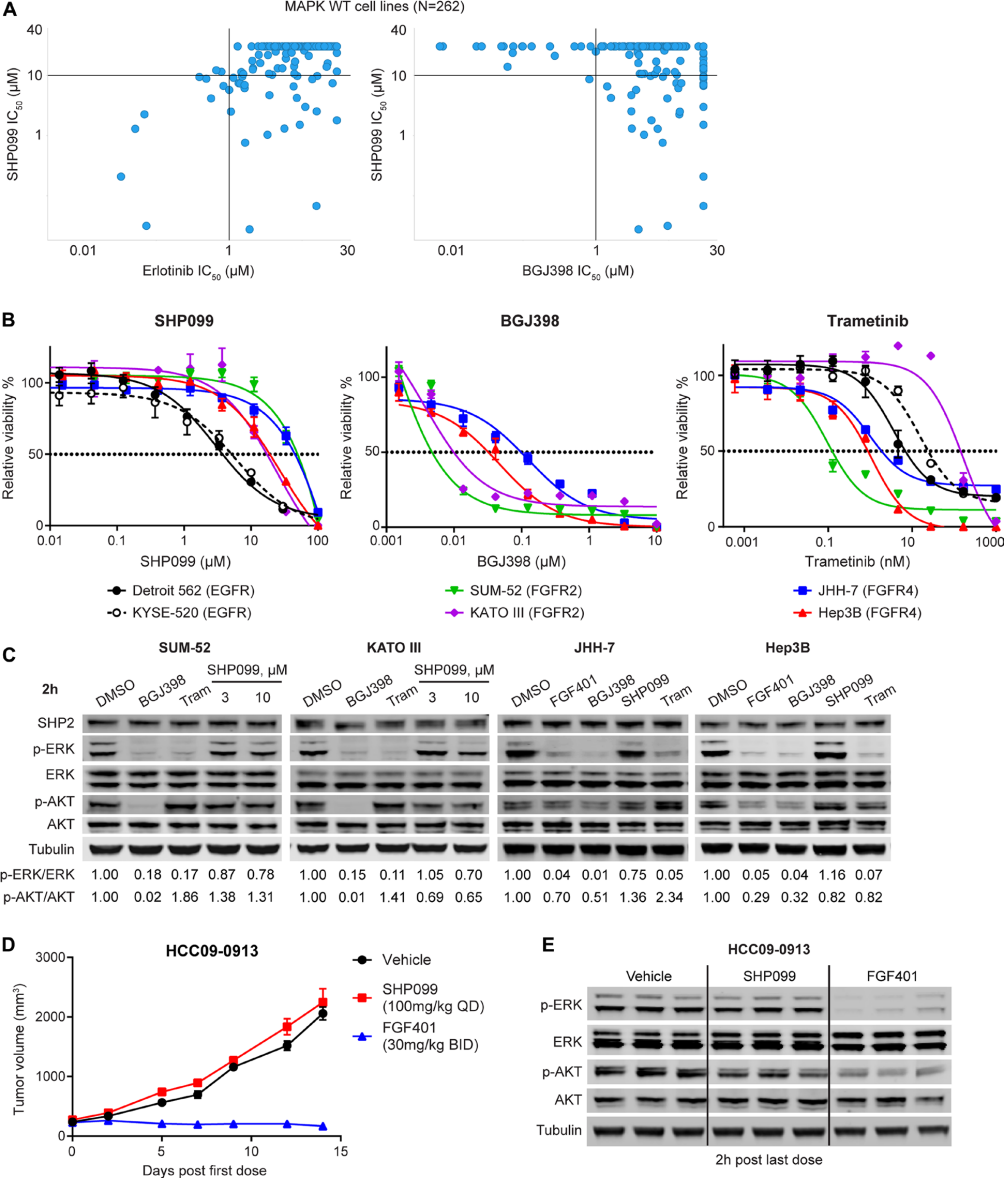
FGFR-driven MAPK-dependent cells are resistant to allosteric SHP2 inhibition. (**A**) Correlation of anti-proliferation IC_50_ values of SHP099 and erlotinib or BGJ398 in 262 MAPK (*KRAS*, *NRAS*, *HRAS*, *BRAF*, and *NF1*) wild-type cell lines generated from high-throughput compound profiling as described [18]. Each dot represents one cell line. (**B**) Anti-proliferative effects of SHP099, BGJ398, or trametinib at the indicated concentrations in a 6-day cell proliferation assay with indicated cell lines (mean percentages of cell viability are shown, error bars denote standard deviation, *n* = 3). (**C**) Immunoblot of SHP2, p-ERK1/2 (T202/Y204), ERK1/2, p-AKT (S473), AKT, and tubulin with indicated cells treated with 0.5 μM BGJ398, 0.1 μM FGF401 for JHH-7 and Hep3B, 10 μM SHP099 (also 3 μM SHP099 for SUM-52 and KATO III), or 0.1 μM trametinib for 2 h. Ratios of p-ERK/ERK and p-AKT/AKT were calculated as described in Materials and Methods. (**D**) Mean tumor volumes of hepatocellular carcinoma patient derived xenograft HCC09-0913 in nude mice following treatment with vehicle, SHP099 (100 mg/kg body weight, daily), or FGF401 (30 mg/kg body weight, twice a day) for 14 days. Error bars, SEM. *n* = 3 mice per group. (**E**) Immunoblot of p-ERK1/2, ERK1/2, p-AKT, AKT, and tubulin from tumor tissues collected at 2 h after last dose from mice treated as described in (D).

We previously demonstrated enrichment for RTK-dependent cell lines within the set of SHP2-dependent cell lines in a pooled shRNA screen performed in a panel of 250 cancer cell lines [10]. To further examine possible RTK-SHP2 dependency correlations, we took advantage of a high-throughput pharmacological profiling of anti-cancer agents that included RTK inhibitors such as erlotinib (EGFR) and BGJ398 (FGFRs) [20] as well as SHP099 (allosteric SHP2 inhibitor) [10], and trametinib (MEK1/2 inhibitor) across 262 cancer cell lines. As cell lines with mutations in genes in the downstream MAPK pathway are often insensitive to RTK inhibition [10], we restricted the analysis to cell lines with wild-type *KRAS*, *NRAS*, *HRAS*, *BRAF*, and *NF1* (Supplementary Table 1). We found that cell lines that are sensitive to erlotinib (IC_50_ < 1 μM, *n* = 10) are all sensitive to SHP099 (IC_50_ < 10 μM) while cell lines that are sensitive to BGJ398 (IC_50_ < 1 μM, *n* = 17) are all resistant to SHP099 (IC_50_ > 10 μM) except Fu97 (Figure 1A; Supplementary Table 1).

In addition to the RAS-MAPK pathway, FGFRs can also activate other effector pathways such as PI3K-AKT and PLCγ-PKC independent of SHP2 [14, 21]. It is possible that the resistance to SHP099 in BGJ398-sensitive cell lines is due to the contribution of non-MAPK pathways to cell growth. Therefore, we used sensitivity to trametinib as a surrogate for the dependency on the MAPK pathway. Nearly half (8 out of 17) of the BGJ398-sensitive cell lines are also sensitive to trametinib (IC_50_ < 0.1 μM) (Supplementary Table 2) and all these eight cell lines were resistant to SHP099, strongly suggesting FGFR-driven MAPK-dependent cells are resistant to allosteric SHP2 inhibition compared with EGFR-driven cells (Figure 1A).

We selected four FGFR-driven cell lines to confirm their resistance to SHP099 in comparison with two EGFR-driven cell lines (KYSE-520 and Detroit 562). SUM-52 and KATO III cells harbor FGFR2 amplification [22] while JHH-7 and Hep3B cells bear FGF19 amplification that activates FGFR4 [23]. In a six-day anti-proliferation assay, all four FGFR-driven cell lines are resistant (IC_50_ > 10 μM) to SHP099 (IC_50_: SUM-52 = 49.62 μM, KATO III = 17.28 μM, JHH-7 = 45.32 μM, and Hep3B = 19.08 μM; Figure 1B) compared with the two EGFR-driven cell lines (IC_50_: Detroit 562 = 3.76 μM and KYSE-520 = 5.14 μM; Figure 1B). This difference in sensitivity was also observed with more potent SHP2 inhibitors including SHP394 [12] and RMC-4550 [24] in JHH-7 and Hep3B compared with Detroit 562 or KYSE-520 (Supplementary Figure 1A). We also verified the dependency of these four FGFR-activated cell lines on FGFR and MAPK using BGJ398 (IC_50_: SUM-52 = 0.005 μM and KATO III = 0.01 μM, JHH-7 = 0.032 μM, and Hep3B = 0.098 μM; Figure 1B) and trametinib (IC_50_: SUM-52 = 0.124 nM, KATO III = 143 nM, JHH-7 = 1.86 nM, and Hep3B = 0.882 nM, KYSE-520 = 7 nM, Detroit 562 = 24 nM; Figure 1B). The slightly weaker efficacy of BGJ398 in the two FGFR4-driven cell lines (JHH-7 and Hep3B) is likely due to its weaker activity against FGFR4 compared with FGFR1-3 [20], and both cell lines were sensitive to FGFR4 specific inhibitor FGF401 [25] (IC_50_: JHH-7 = 0.015 μM, and Hep3B = 0.036 μM; Supplementary Figure 1A). Consistent with the lack of anti-proliferative effect, there was little reduction (no more than 30% reduction of p-ERK levels by 10 μM SHP099 in all four cell lines; Figure 1C) in p-ERK levels following a two-hour treatment with SHP099 while all four cell lines express SHP2. As expected, both 0.5 μM BGJ398 and 0.1 μM trametinib fully inhibited ERK phosphorylation in these four FGFR-driven cell lines (Figure 1C). In all FGFR-driven cell lines except JHH-7, treatment with FGFR inhibitors also reduced phospho-AKT levels, raising the possibility that lack of SHP099 efficacy could be partially attributed to its lack of effect on p-ATK levels (less than 20% reduction by 10 μM SHP099 except in KATO III cells; Figure 1C). However, addition of alpelisib (BYL719), a PI3Kα specific inhibitor [26], did not greatly sensitize these FGFR-driven cells to SHP394 (Supplementary Figure 1B), suggesting that the FGFR-activated PI3K activity did not significantly contribute to the resistance to SHP2 inhibitors.

We also evaluated the *in vivo* efficacy of SHP099 on an FGFR4-dependent patient-derived hepatocellular carcinoma xenograft HCC09-0913. Consistent with the findings *in vitro*, SHP099 at its maximum tolerated dose of 100 mg per kilogram bodyweight (mpk) daily (QD) had no anti-tumor effect while the FGFR4 inhibitor FGF401 [25] dosed at 30 mpk twice every day (BID) effectively suppressed tumor growth (Figure 1D). Consistent with the efficacy results, SHP099 had no effect on p-ERK levels in tumors harvested at the end of the study while FGF401 strongly inhibited ERK phosphorylation (Figure 1E). Interestingly, p-AKT levels were modestly reduced by SHP099 in the tumors, which did not translate to any anti-tumor efficacy (Figure 1D, 1E).

### FGFR-driven MAPK signaling and growth depend on SHP2

**Figure 2:**
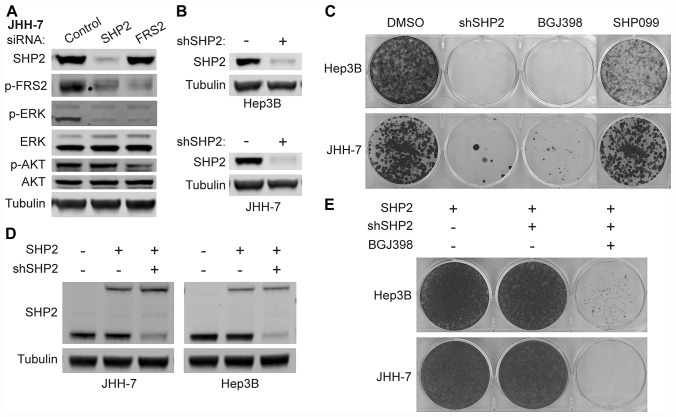
FGFR-activated MAPK signaling depends on SHP2. Immunoblot of indicated proteins in JHH-7 cells transfected with siRNAs targeting SHP2 or FRS2 or scrambled control siRNA for 3 days (**A**) or Hep3B and JHH-7 cells stably expressing doxycycline-inducible shRNA targeting SHP2 (shSHP2) treated with PBS (-) or 100 ng/ml doxycycline (+) for 4 days (**B**). (**C**) Colony formation assay with cells described in (B) treated with DMSO, 100 ng/ml doxycycline (shSHP2), 0.1 μM BGJ398, or 10 μM SHP099 for 10–14 days, followed by crystal violet staining. (**D**) Immunoblot of SHP2 and tubulin with cells described in (B) that were further engineered to constitutively express shSHP2-resistant SHP2 (with a biotin ligase tag, upper band) and parental JHH-7 or Hep3B cells as a control (first lane). (**E**) Colony formation assay with cells described in (D) treated with DMSO, 100 ng/ml doxycycline (+shSHP2), or doxycycline plus 0.1 μM BGJ398 for 14–21 days, followed by crystal violet staining.

To investigate whether SHP2 is dispensable for FGFR-activated MAPK signaling, we used siRNAs to knock down the expression of either SHP2 or FRS2 in JHH-7 and Hep3B cells, achieving more than 90% knockdown on the mRNA levels for both genes (Supplementary Figure 1C). As shown in Figure 2A, both SHP2 and FRS2 knockdown reduced p-ERK levels in JHH-7 cells. Similar observations were also made in Hep3B cells (Supplementary Figure 1D). In addition, SHP2 knockdown also reduced phospho-FRS2 (p-FRS2; Y436) levels (Figure 2A; Supplementary Figure 1D), an activation marker of FGFRs, suggesting SHP2 may directly mediate the activation of the adaptor complex. Loss of SHP2 did not affect p-AKT levels in either JHH-7 or Hep3B cells while knockdown of FRS2, the substrate of FGFR, similar to FGFR inhibitor treatment (Figure 1C), reduced p-AKT levels (Figure 2A; Supplementary Figure 1D). These data suggest that the PI3K signaling in these cells may be directly activated by FGFR4 independent of SHP2. We next generated JHH-7 and Hep3B cells stably expressing doxycycline-inducible shRNA targeting SHP2 to evaluate their growth dependency on SHP2 as SUM-52 and KATO III both have been shown to be dependent on SHP2 for cell proliferation [10]. Four days of doxycycline treatment led to near complete knockdown of SHP2 in both cell lines (Figure 2B). In a two-week colony formation assay, SHP2 knockdown, but not 10 μM SHP099 treatment, effectively suppressed cell growth of JHH-7 and Hep3B, similar to 0.1 μM BGJ398 treatment (Figure 2C). Importantly, constitutive expression of a shRNA-resistant SHP2 with a biotin ligase tag at levels comparable to endogenous SHP2 in both cell lines (Figure 2D) rescued the growth suppression by SHP2 knockdown shown in Figure 2C while the cells remained sensitive to BGJ398 (Figure 2E), suggesting growth suppression by SHP2 shRNA in both Hep3B and JHH-7 cells is due to on-target knockdown of SHP2.

We also correlated the genetic dependencies on FRS2 and SHP2 in a pooled shRNA viability screen from Project DRIVE [27] and found that most cell lines that are dependent on FRS2 (ATARiS score < −0.5, top 5%) displayed certain degrees of SHP2 dependency (ATARiS score < −0.25) (Supplementary Figure 1E), which is in line with EGFR dependent cell lines (ATARiS score < −1) as previously defined [27]. Taken together, these data indicate that FGFR-driven MAPK pathway activation and cell proliferation depends on SHP2, despite the observed resistance to allosteric inhibition.

### Rapid feedback activation of FGFR causes resistance to SHP2 inhibitors

**Figure 3:**
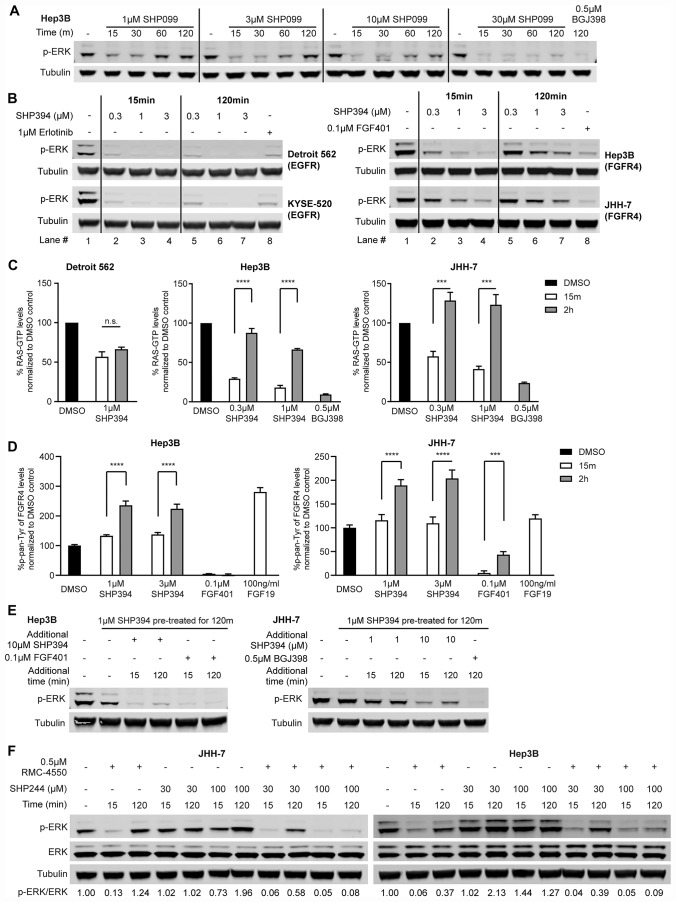
Rapid feedback activation of FGFR causes resistance to SHP2 inhibitors. (**A**) Immunoblot of p-ERK in Hep3B cells treated with SHP099 at indicated concentrations (1 μM, 3 μM, 10 μM, or 30 μM) for 15 min, 30 min, 60 min, or 120 min, or 0.5 μM BGJ398 for 2 h. (**B**) Immunoblot of p-ERK and tubulin in Detroit 562, KYSE-520, Hep3B, and JHH-7 cells treated with either SHP394 at indicated concentrations (0.3 μM, 1 μM, or 3 μM) for 15 min or 2 h or an RTK inhibitor (1 μM erlotinib for Detroit 562 and KYSE-520; 0.1 μM FGF401 for JHH-7 and Hep3B) for 2 h. Lane numbers were added for the convenience of comparison. (**C**) Detroit 562 cells were treated with 1 μM SHP394 for 15 min or 2 h and Hep3B and JHH-7 cells were treated with either SHP394 at indicated concentrations (0.3 μM or 1 μM) for 15 min or 2 h or 0.5 μM BGJ398 for 2 h. RAS-GTP levels were determined by a G-LISA RAS activation assay. n. s., not significant; ^***^, *p* < 0.001; ^****^, *p* < 0.0001 (by t-test). (**D**) Hep3B and JHH-7 cells were treated with SHP394 at indicated concentrations (1 μM or 3 μM) or 0.1 μM FGF401 for 15 min or 2 h or 100 ng/ml FGF19 for 15 min. Pan-tyrosine phosphorylation levels of FGFR4 were determined by the Phospho-FGF Receptor 4 (panTyr) Sandwich ELISA assay. ^***^, *p* < 0.001; ^****^, *p* < 0.0001 (by *t*-test). (**E**) Hep3B and JHH-7 cells were pre-treated with 1 μM SHP394 for 2 h to induce the rebound of p-ERK levels, followed by addition of 10 μM SHP394 (also 1 μM SHP394 treatment for JHH-7), 0.1 μM FGF401 for Hep3B, or 0.5 μM BGJ398 for JHH-7 for either 15 min or 2 h. Cell lysates were collected and p-ERK1/2 levels were determined by immunoblotting. (**F**) Immunoblot of p-ERK and tubulin in JHH-7 and Hep3B cells treated with 0.5 μM RMC-4550 (site 1 inhibitor), site 2 inhibitor SHP244 (30 or 100 μM), or the combination of RMC-4550 and SHP244 for either 15 min or 2 h. Ratios of p-ERK/ERK were calculated as described in Materials and Methods.

To investigate the disconnect between genetic dependency on SHP2 and the resistance to pharmacological SHP2 inhibition in FGFR-driven cells, we performed a time-course analysis of p-ERK levels in Hep3B cells treated with a range of concentrations of SHP099. Surprisingly, at early time points (15 min and 30 min), SHP099, even at low concentrations such as 1 μM and 3 μM, effectively reduced p-ERK levels. However, levels of p-ERK rebounded within one to two hours (Figure 3A). Higher concentrations of SHP099 delayed (10 μM) or prevented (30 μM) this p-ERK rebound within two hours and ERK phosphorylation was also inhibited after two-hour treatment with 0.5 μM BGJ398 (Figure 3A). Similarly, p-MEK levels in Hep3B were also reduced by 1 μM SHP099 within the first 30 minutes of treatment and rebounded by two hours, which was prevented by a higher concentration (30 μM) of SHP099 (Supplementary Figure 2A). In JHH-7 cells, SHP099 concentrations as high as 30 μM failed to prevent the p-ERK rebound whereas two-hour treatment with either 0.5 μM BGJ398 or 0.1 μM FGF401 effectively suppressed p-ERK levels (Supplementary Figure 2B).

This rapid p-ERK rebound following SHP099 treatment was also confirmed using a more quantitative Meso Scale Discovery (MSD) p-ERK assay in two additional FGFR-driven cell lines (SUM-52 and ECC10) as well as in Hep3B and JHH-7 (Supplementary Figure 2C). In all four cell lines, the p-ERK rebound could be prevented or reduced by up to 100 μM SHP099 treatment (Supplementary Figure 2C). Similar two-hour p-ERK rebound was also observed when Hep3B and JHH-7 cells were treated with SHP394 (Lane 2 vs 5, Lane 3 vs. 6, and Lane 4 vs. 7), while there was no p-ERK rebound in the two EGFR-driven cell lines when treated with SHP394 with a concentration as low as 0.3 μM (Figure 3B). And the lack of the p-AKT modulation by SHP2 inhibitor in JHH-7 and Hep3B cells (Figure 1C) was not due to a rebound since p-AKT levels in both cell lines were not affected by SHP394 treatment even at 15 minutes (Supplementary Figure 2D). In addition, the p-ERK rebound was also observed with RMC-4550 while FGFR inhibitors maintained ERK inhibition throughout the two-hour treatment in all four FGFR-driven cell lines tested (Supplementary Figure 2E). Taken together, these data suggest FGFR-activated SHP2 is inhibited by allosteric inhibition initially but there is rapid feedback activation of ERK, limiting its activity.

The RAS-MAPK pathway is tightly modulated through feedback regulation at multiple nodes of the pathway (e. g. RTK, RAS, RAF, MEK and ERK) to control ERK activity output [28]. The p-MEK rebound in parallel with the p-ERK rebound following SHP099 treatment (Figure 3A; Supplementary Figure 2A) suggests the rapid ERK reactivation is likely from the upstream regulation rather than direct regulation on ERK itself through DUSP and/or others. To pinpoint the source of this rapid p-ERK rebound, we first examined whether RAS was reactivated when p-ERK levels rebounded following SHP2 inhibitor treatment by measuring the RAS-GTP levels using a G-LISA RAS activation assay (Figure 3C). In EGFR-driven Detroit 562 cells, SHP394 treatment reduced RAS-GTP levels at 15-minute treatment and maintained the suppression within two hours (Figure 3C). Similar to the dynamics of p-ERK levels, in both JHH-7 and Hep3B cells, the RAS-GTP levels were first reduced by SHP394 treatment at 15 min but quickly rebounded within two hours while BGJ398 effectively reduced RAS-GTP levels two hours post treatment (Figure 3C). To further ascertain whether this RAS re-activation was caused by FGFR feedback activation, we performed an ELISA assay measuring the pan-tyrosine phosphorylation levels of FGFR4 (p-FGFR4) due to the lack of a robust p-FGFR4 antibody for immunoblot. In Hep3B cells, treatment with SHP394 had no effect on p-FGFR4 at 15 min as expected but caused an increase of p-FGFR4 levels at two hours that was similar to the effect of 15-min stimulation by FGF19 treatment, the ligand of FGFR4 (Figure 3D). In contrast, treatment with FGF401 effectively inhibited p-FGFR4 throughout the two-hour treatment (Figure 3D). Similar observation was also made in JHH-7 cells with the exception that there is a slight but statistically significant p-FGFR4 rebound during FGF401 treatment and the FGF19 treatment failed to further stimulate FGFR4 (Figure 3D). Total FGFR4 levels were not affected by SHP394 treatment in both Hep3B and JHH-7 cells (Supplementary Figure 2D). In contrast, SHP394 treatment did not cause an increase of phospho-EGFR at Y1068 (p-EGFR) levels as EGF treatment did in Detroit 562 cells (Supplementary Figure 2F), suggesting EGFR was not rapidly feedback activated, which is consistent with the sustained inhibition of ERK activity by SHP2 inhibitors. These data collectively suggest that the rapid p-ERK rebound following SHP2 inhibitor treatment in FGFR-driven cells is likely due to the feedback activation of FGFRs and RAS.

Next we sought to investigate whether this RAS reactivation following initial SHP2 inhibition is dependent on SHP2. We first treated Hep3B and JHH-7 cells with 1 μM SHP394 for two hours to induce the p-ERK rebound and then added additional SHP394 as indicated for either 15 minutes or two hours (Figure 3E). Addition of 10 μM SHP394 suppressed the rebound of p-ERK levels at 15 min and the suppression was maintained throughout the two-hour treatment despite a slight rebound in both cell lines. In contrast, addition of 1 μM SHP394 had limited effect on the rebounded p-ERK in JHH-7 cells (Figure 3E). SHP394-induced p-ERK rebound was effectively abolished by two-hour treatment with either FGF401 in Hep3B or BGJ398 in JHH-7 (Figure 3E).

SHP2 can cycle between an open active conformation and a closed inactive conformation, in which the catalytic site is hindered by the N-terminal SH2 domain. The above referenced allosteric SHP2 inhibitors such as SHP099, SHP394, and RMC-4550 bind to the tunnel-like pocket formed by the confluence of three domains of SHP2 (site 1 inhibitor) and stabilize the closed auto-inhibited state of SHP2. We also reported a series of site 2 allosteric inhibitors such as SHP244 that bind to a distinct latch-like allosteric site in a cleft at the interface of the N-terminal SH2 and PTP domains [29]. We also demonstrated that site 1 and site 2 inhibitors can simultaneously bind to SHP2 and cooperatively stabilize the closed inactive conformation of SHP2 [29]. Therefore, we tested whether the combination of site 1 and site 2 SHP2 inhibitors is effective at inhibiting ERK activity in the FGFR-driven cells resistant to site 1 SHP2 inhibitors. Due to the low cellular potency of SHP244, we treated JHH-7 and Hep3B cells with 30 or 100 μM SHP244 alone to test the single agent effect of the site 2 inhibitor on the p-ERK levels and then we combined site 1 inhibitor RMC-4550 and SHP244 and treated the cells for either 15 minutes or two hours to test whether the addition of SHP244 can prevent the rebound of p-ERK levels following the initial reduction by RMC-4550 treatment (Figure 3F). Consistent with the transient ERK inhibition by RMC-4550 in JHH-7 and Hep3B cells (Supplementary Figure 2E), up to 100 μM SHP244 alone had little effect on ERK phosphorylation following either 15-minute or two-hour treatment. However, SHP244 treatment effectively prevented the RMC-4550-induced p-ERK rebound (more than 75% reduction of the rebound of p-ERK levels by 100 μM SHP244) throughout the two-hour treatment in both cell lines (Figure 3F). Moreover, the addition of SHP244 to RMC-4550 appeared more effective inhibiting ERK phosphorylation at two hours post treatment than 20-fold higher dose (10 μM) of RMC-4550 (Figure 3F; Supplementary Figure 2E). We also investigated whether inhibition of SOS1 and RAS interaction using BAY-293 [30], can prevent the SHP2 inhibitor-induced feedback activation of ERK. Similar to SHP244, BAY-293 alone had little effect on p-ERK levels but it effectively abolished the rebound of p-ERK levels following two-hour treatment with RMC-4550 (Supplementary Figure 2G). In addition, phosphorylation of SHP2 (p-SHP2) at Y542 and Y580 at its C-terminal tail is often increased by RTK activation and sometimes associated with increased SHP2 activity [9, 31]. Interestingly, we observed a strong p-SHP2 induction two hours after RMC-4550 treatment in both JHH-7 and Hep3B cells (Supplementary Figure 2H), suggesting an increased RTK activity likely from FGFR4, which is consistent with the increase of p-FGFR4 levels as described earlier (Figure 3D). These data suggest that SHP2 inhibition in certain FGFR-driven cell lines may cause rapid feedback activation of FGFRs and further activation of SHP2, which requires either a much higher dose of site 1 allosteric inhibitors or the combination of site 1 and 2 inhibitors to block its activity.

### MEKi-induced FGFR-mediated feedback activation of SHP2 is also resistant to allosteric inhibitors

**Figure 4:**
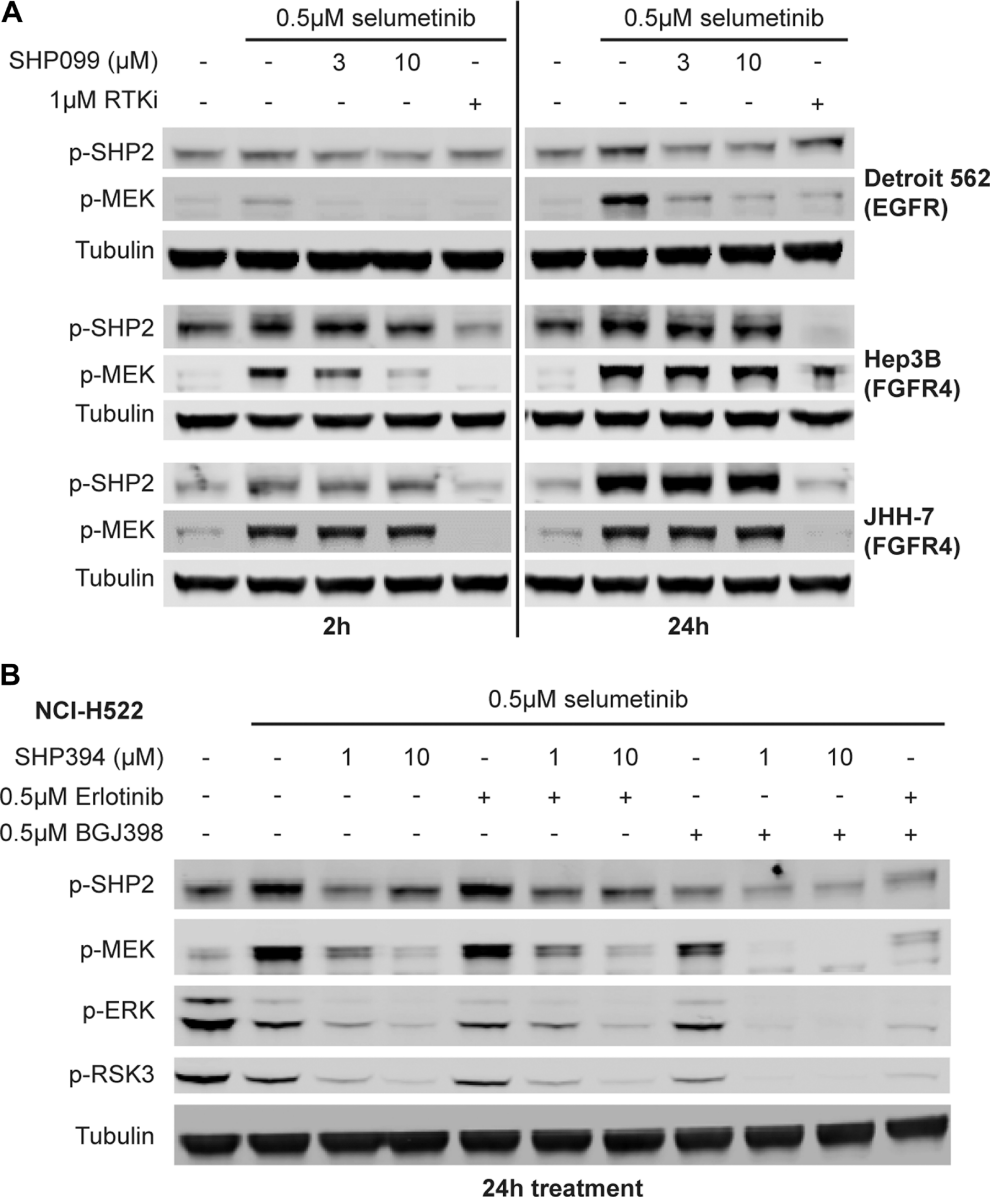
MEKi-induced feedback activated SHP2 by FGFR is resistant to allosteric SHP2 inhibitors. (**A**) Immunoblot of p-SHP2 (Y542), p-MEK1/2 (S217/221), and tubulin in Detroit 562, Hep3B and JHH-7 cells treated with 0.5 μM selumetinib alone or in combination with 3 or 10 μM SHP099 or an RTK inhibitor (1 μM erlotinib for Detroit 562 and 1 μM BGJ398 for the rest) for either 2 h or 24 h. (**B**) NCI-H522 cells were treated with 0.5 μM selumetinib alone or in combination with 1 or 10 μM SHP394, 0.5 μM erlotinib, 0.5 μM BGJ398, SHP394 plus erlotinib, SHP394 plus BGJ398, or erlotinib plus BGJ398. Cell lysates were collected and p-SHP2, p-MEK1/2, p-ERK1/2, and p-RSK3 (T356/S360) levels were determined by immunoblotting.

To further investigate whether this SHP2 inhibitor-resistant rapid feedback activation of SHP2 by FGFRs is a unique phenomenon due to the binding of allosteric inhibitors to SHP2 or a general effect due to ERK inhibition, we used the MEK inhibitor selumetinib to trigger the pathway feedback activation as previously described [32, 33]. In Detroit 562 cells, a robust p-MEK induction was not observed at 2 h but until 24 h following treatment with 0.5 μM selumetinib and could be prevented by SHP099 at a concentration as low as 3 μM or by 1 μM erlotinib (Figure 4A). However, in JHH-7 cells, a robust p-MEK induction was detected as early as two hours after selumetinib treatment and up to 10 μM SHP099 could not prevent the p-MEK induction at either 2 h or 24 h time point, yet it was effectively suppressed by 1 μM BGJ398 (Figure 4A). A similar observation was also made in Hep3B cells (Figure 4A) and another FGFR-driven cell line ECC10 (Supplementary Figure 3A) except that only 10 μM but not 3 μM SHP099 blocked p-MEK induction at the two-hour time point (Figure 4A). Interestingly, the phospho-SHP2 (Y542) levels in all three FGFR-driven cell lines were also elevated just after two-hour treatment with selumetinib, which became more evident at 24 h and could not be prevented by co-treatment with up to 10 μM SHP099 (Figure 4A), suggesting a strong SHP2 activation likely by FGFRs. This p-SHP2 induction was not observed in Detroit 562 cells at 2 h and was modest at 24 h following selumetinib treatment (Figure 4A).

To compare the differential sensitivity of FGFR- and EGFR-activated SHP2 to allosteric inhibitors in an isogenic background, we identified a cell line NCI-H522 in which both EGFR and FGFR mediate the selumetinib-induced feedback activation of MEK (Supplementary Figure 3B). When the feedback activation was driven predominantly by FGFR following EGFR inhibition by co-treatment with 0.5 μM erlotinib, 1 μM SHP394 treatment only partially prevented the adaptive p-MEK induction following 0.5 μM selumetinib treatment for 24 h, which was only blocked by 10 μM SHP394 treatment (Figure 4B). In contrast, when the feedback activation was driven by EGFR following FGFR inhibition by co-treatment with 0.5 μM BGJ398, 1 μM SHP394 treatment effectively blocked p-MEK induction (Figure 4B). Again, selumetinib treatment led to upregulation of p-SHP2, which could be prevented by FGFR inhibition but not EGFR inhibition (Figure 4B), suggesting a possibly greater activating effect on SHP2 from FGFR activity compared with EGFR. Taken together, these data suggest FGFRs may have a rapid feedback activation mechanism upon ERK inhibition that leads to increased SHP2 phosphorylation and resistance to allosteric SHP2 inhibitors.

### Rapid FGFR feedback activation is caused by downregulation of Sprouty proteins

**Figure 5:**
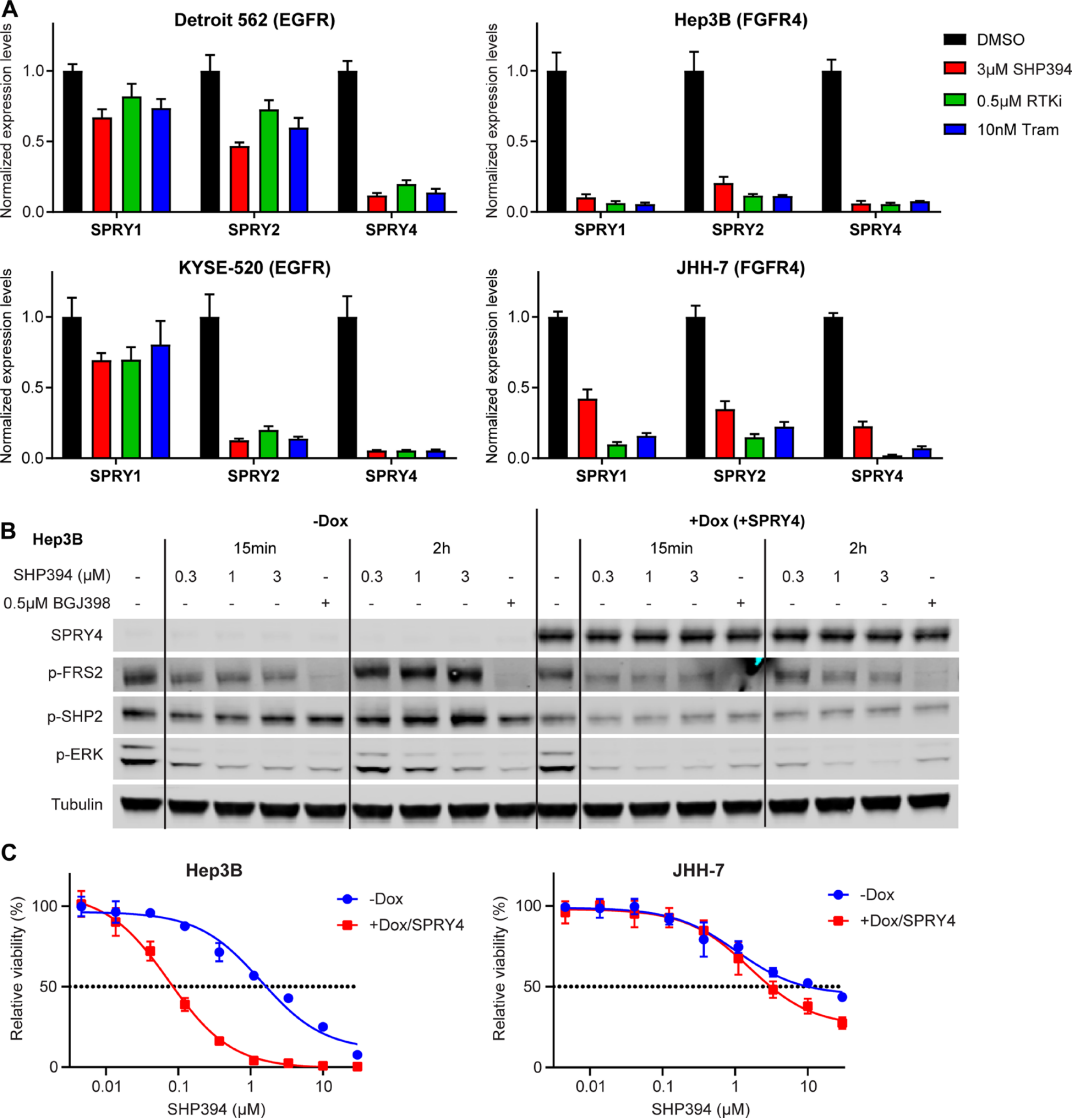
Rapid FGFR feedback activation is caused by downregulation of Sprouty proteins. (**A**) mRNA levels determined by qRT-PCR of *SPRY1*, *SPRY2*, and *SPRY4* in Detroit 562, KYSE-520, Hep3B, and JHH-7 cells treated with DMSO, 3 μM SHP394, 0.5 μM RTK inhibitor (erlotinib for Detroit 562 and KYSE-520, BGJ398 for Hep3B and JHH-7), or 10 nM trametinib for 2 h. Statistical analysis (paired t-tests) was described in Supplementary Table 3. (**B**) Hep3B cells stably expressing doxycycline-inducible SPRY4 were treated with PBS (−Dox) or 100 ng/ml doxycycline (+SPRY4) for 48 h, followed by treatment with DMSO, SHP394 at indicated concentrations, or 0.5 μM BGJ398 for either 15 min or 2 h. Cell lysates were collected and p-FRS2 (Y436), p-SHP2 (Y542), p-ERK1/2, and SPRY4 levels were determined by immunoblotting. (**C**) Anti-proliferative effects of SHP394 at the indicated concentrations in a 6-day cell proliferation assay with Hep3B and JHH-7 cells stably expressing doxycycline-inducible SPRY4 in the absence (−Dox) or presence (+Dox/SPRY4) of 100 ng/ml doxycycline (mean percentages of cell viability are shown; error bars, SD; *n* = 3).

We next investigated the mechanism of rapid FGFR feedback activation described above. Sprouty family proteins (SPRYs), consisting of SPRY1-4, have been reported to negatively regulate RTK activity by sequestering the GRB2-SOS1 complex and their expression is regulated by ERK signaling [34, 35]. Based on gene expression levels determined by RNAseq, *SPRY1/2/4* collectively were expressed at higher levels in the five FGFR-driven cell lines than in the three EGFR-driven lines, with undetectable expression of *SPRY3* in all cell lines tested (Supplementary Figure 4A). We then compared *SPRY1/2/4* modulation by inhibitors of SHP2, FGFR, and MEK after two-hour treatment in FGFR-driven cells with that in EGFR-driven cells. The *SPRY1/2/4* transcript levels were drastically decreased by all inhibitors in Hep3B, JHH-7 (Figure 5A; Supplementary Table 3), SUM-52, and ECC10 (Supplementary Figure 4B) while the decrease was not statistically significant in Detroit 562 and KYSE-520 cells (Figure 5A; Supplementary Table 3) and another EGFR-driven cell line HCC827 (Supplementary Figure 4B). Interestingly, FGFR-driven cells tend to express higher levels of SPRYs (Supplementary Figure 4A). Taken together with the more rapid SPRY downregulation upon ERK inhibition than EGFR-driven cell lines, these data suggest that FGFRs may be tightly regulated by SPRYs.

Next we tested whether Sprouty proteins can block the feedback activation of FGFR following SHP2 inhibition in FGFR-driven cells. A doxycycline-inducible SPRY4 was introduced into JHH-7 and Hep3B cells. SPRY4 over-expression was detected as early as 6 h after doxycycline treatment and caused a modest decrease of p-ERK levels in both JHH-7 and Hep3B (Supplementary Figure 5A). SPRY4 over-expression prevented the p-ERK rebound after two-hour treatment with low concentrations of SHP394 (0.3 μM and 1 μM) in Hep3B cells, comparable to the effects achieved by either 3 μM SHP394 or 0.5 μM BGJ398 (Figure 5B). Consistently, SPRY4 over-expression also markedly sensitized Hep3B cells to SHP394 by nearly 20 fold (IC_50_: −Dox = 1.64 μM, +Dox/SPRY4 = 0.08 μM; Figure 5C). Similar effects on p-ERK levels by SPRY4 over-expression were also observed in JHH-7 cells (Supplementary Figure 5B), which led to a 3-fold sensitization to SHP394 (IC_50_: −Dox = 10.10 μM, +Dox/SPRY4 = 3.10 μM; Figure 5C). Similar sensitization by SPRY4 over-expression was also observed with RMC-4550 (Supplementary Figure 5C). In addition, possibly consistent with the function of SPRY4, the increases in p-FRS2 and p-SHP2 levels following two-hour SHP394 treatment, likely a result of FGFR feedback activation (Figure 3D), were effectively blocked by SPRY4 overexpression in Hep3B cells (Figure 5B), but not in JHH-7 cells (Supplementary Figure 5B). This difference may explain the relatively modest sensitization to SHP2 inhibitors by SPRY4 over-expression in JHH-7 compared with Hep3B cells (Figure 5C; Supplementary Figure 5C). These data suggest that the rapid FGFR feedback activation upon SHP2 inhibition may be due to the downregulation of pathway negative regulators such as SPRYs.

### FGFR and SHP2 inhibitors exhibit combination synergy inhibiting FGFR-driven cell proliferation

**Figure 6:**
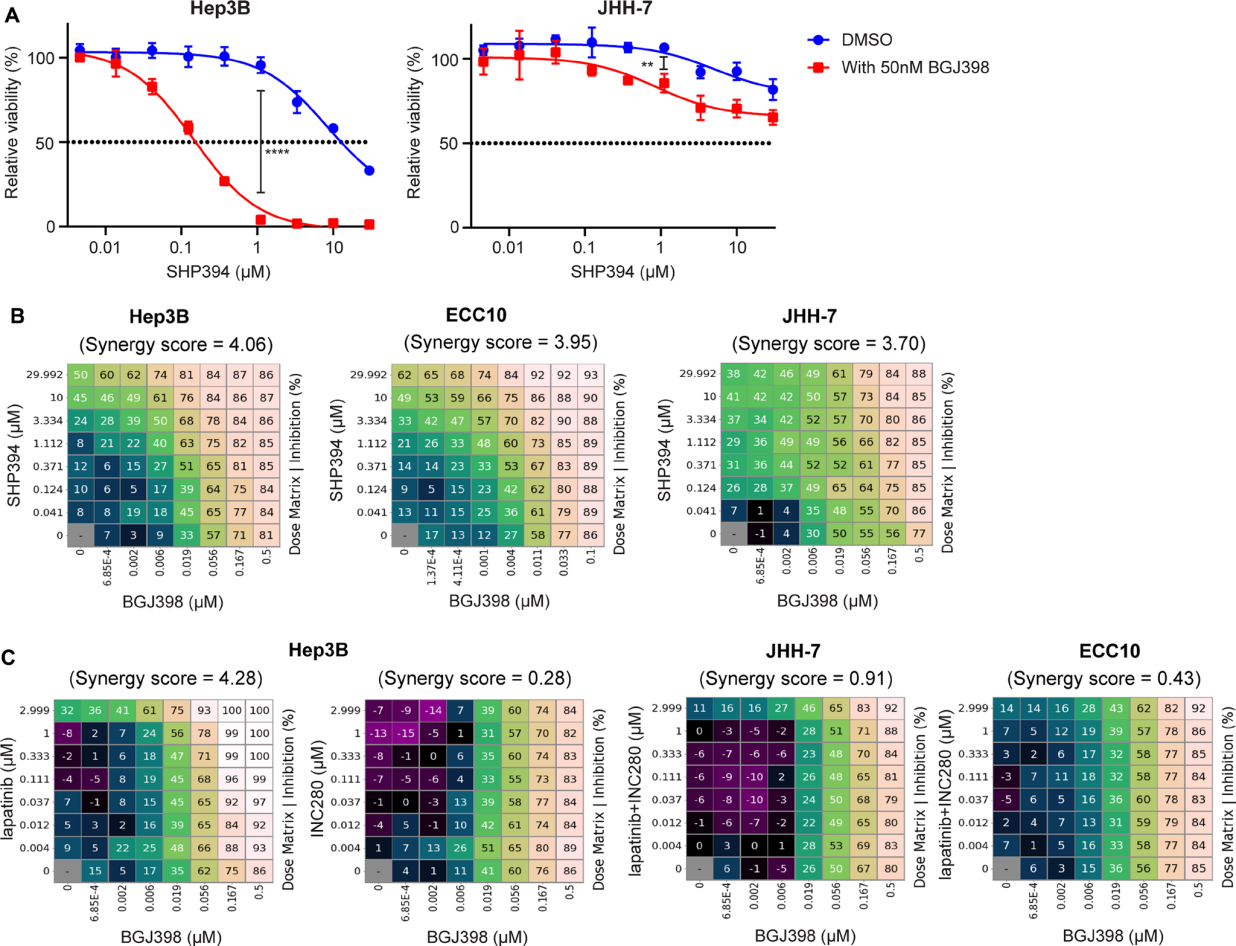
FGFR and SHP2 inhibitors exhibit synergy inhibiting FGFR-driven cell growth. (**A**) Anti-proliferative effects of SHP394 at the indicated concentrations in a 3-day cell proliferation assay with Hep3B and JHH-7 cells in the absence or the presence of 50 nM BGJ398 (mean percentages of cell viability are shown; error bars, SD; *n* = 3) ^**^, *p* < 0.01; ^****^, *p* < 0.0001 (by t-test). (**B**) Hep3B, ECC10, and JHH-7 cells were treated with BGJ398 and SHP394 in dose matrix for 6 days. (**C**) Hep3B cells were treated with BGJ398 and lapatinib or INC280 in dose matrix for 6 days while JHH-7 and ECC10 cells were treated with BGJ398 and the combination of lapatinib plus INC280 in dose matrix for 6 days. The mean (*n* = 3) of percentages of inhibition (relative to the DMSO-treated control) of compound-treated cells and synergy scores between the two compounds were determined using the Combination Analysis Module as previously reported [36]. Synergy scores greater than 2 are considered to have synergistic rather than additive effects.

Since no pharmacological agents increasing SPRY expression or activity are available, we examined whether FGFR inhibition, which prevents the assembly of the FGFR-activated adaptor complex recruited and achieves similar effects as SPRY overexpression, can sensitize these FGFR-driven cells to SHP2 inhibitors. Indeed, BGJ398 sensitized Hep3B and, to a less extent, JHH-7 cells to SHP394 in a 3-day cell viability assay at a concentration (50nM) with minor single agent effect (29% growth inhibition for Hep3B and 18% for JHH-7; Figure 6A). Consistent with SPRY4 over-expression (Figure 5C), the sensitization to SHP394 by BGJ398 co-treatment in JHH-7 was relatively modest compared with Hep3B (Figure 6A).

We then investigated whether SHP2 and FGFR inhibitors may have synergistic effect inhibiting FGFR-driven cell proliferation using a dose matrix combination assay in which synergy score higher than 2 is considered synergistic [36]. BGJ398 and SHP394 exhibited a strong synergy inhibiting cell proliferation in all three FGFR-driven cell lines tested in a 6-day cell viability assay (synergy score: Hep3B = 4.06, ECC10 = 3.95, JHH-7 = 3.70; Figure 6B). Given that SHP2 inhibitors were not effective at inhibiting FGFR signaling in those cells as shown in this study, this is possibly due to the feedback activation of other RTK(s) following BGJ398 treatment. We further ascertained which RTK is activated resulting from FGFR inhibition using individual RTK inhibitors and found that EGFR/HER2 dual inhibitor lapatinib, similar to SHP394, but not MET inhibitor INC280 had a strong synergy with BGJ398 (Figure 6C), suggesting ERBB family proteins were feedback activated following FGFR inhibition in Hep3B cells. Similarly, a strong synergy between SHP394 and BGJ398 was also observed in JHH-7 and ECC10 cells (Figure 6C). However, we could not identify the exact feedback activated RTK(s) since there was little synergy when combining lapatinib and INC280 with BGJ398 (Figure 6C), suggesting RTK(s) other than ERBBs and MET or other SHP2 activation mechanisms were involved in JHH-7 and ECC10 cells. Taken together, these data suggest that SHP2 inhibitors may overcome adaptive resistance to FGFR inhibitors possibly due to activation of other RTKs or other SHP2 activation mechanisms in FGFR-driven cancers.

## DISCUSSION

We found that FGFR-dependent cell lines were unexpectedly resistant to several structurally distinct allosteric SHP2 inhibitors compared with EGFR-dependent cell lines in both growth assays and in MAPK pathway modulation *in vitro* and *in vivo* (Figure 1; Supplementary Figure 1A). We first hypothesized that there could be a SHP2 independent mechanism through which FGFR activates RAS or directly activates the MAPK pathway, as suggested by Ahmed *et al.* [13]. However, on-target knockdown of SHP2 using siRNA or shRNA suppressed the MAPK signaling and cell growth in FGFR4-dependent JHH-7 and Hep3B cells (Figure 2A–2C; Supplementary Figure 1D), suggesting that FGFR-driven cells are dependent on SHP2 but resistant to allosteric inhibitors. We then examined the SHP2 dependency of feedback activation in BRAF mutant thyroid cancer cell line SW1736 used in the reported study [13]. We found that co-treatment with both BGJ398 and high dose (10 μM) of RMC-4550 was able to prevent the p-ERK rebound following 24-hour treatment with vemurafenib (Supplementary Figure 6A), indicating that the feedback activation in SW1736 is still dependent on SHP2 but rather resistant to allosteric inhibition. A more detailed profiling of the effect of SHP2 inhibitors on p-ERK levels in FGFR-driven cells revealed that even low concentrations of SHP2 inhibitors (e. g. 1 μM SHP099) could initially reduce p-ERK levels, which rapidly and fully rebounded within two hours, a kinetics that has never been reported before. We further demonstrated that this p-ERK rebound was a result of rapid feedback activation of FGFRs through SHP2 and RAS (Figure 3C, 3E). In addition, this rapid feedback activation of FGFRs and resistance to SHP2 inhibitors was also observed upon ERK inhibition using MEK inhibitors (Figure 4) and is likely through the downregulation of SPRYs (Figure 5A; Supplementary Figure 4B). The differences in the regulation of SPRYs between FGFR- and EGFR-driven backgrounds remain to be confirmed in more cell lines.

The rapid FGFR feedback activation is likely also triggered by FGFR inhibition since SPRYs were also downregulated following FGFR inhibitor treatment similar to SHP2 inhibitors (Figure 5A; Supplementary Figure 4B). Indeed, there was still a small but significant rebound of p-FGFR4 levels in JHH7 cells treated with FGF401 for 2 h (Figure 3D) and consistent with the observation, JHH-7 appeared to be more resistant to SHP2 inhibitors compared with other FGFR-driven cells (Figure 1B; Supplementary Figure 1A). However, the feedback activation did not affect the efficacy of FGFR inhibitors as it did with SHP2 inhibitors. Both FGF401 and BGJ398 maintained ERK inhibition throughout two-hour treatment in all four FGFR-driven cell lines (Supplementary Figure 2E). We hypothesize that this may be due to the distinct mechanisms of action between FGFR inhibitors and allosteric SHP2 inhibitors. BGJ398 and FGF401 belong to ATP-competitive inhibitors that are often designed to potently inhibit activated conformation of kinases, especially those type 1 inhibitors [37]. Therefore, the feedback activation of FGFRs might only modestly shift the potency of FGFR inhibitors. Allosteric SHP2 inhibitors, however, bind to a tunnel-like pocket on SHP2 as a molecular glue, stabilizing the closed autoinhibitory conformation and delaying the conversion to the open and active conformation driven by RTK activation [11, 12]. Conceivably, the rapid feedback activation of SHP2 by FGFRs as evidenced by p-SHP2 induction (Supplementary Figure 2H) may render the allosteric inhibitors ineffective through increasing the pool of open conformation SHP2. This ineffectiveness may be overcome by the addition of site 2 allosteric SHP2 inhibitors such as SHP244, which bind to a distinct latch-like allosteric site simultaneously with site 1 inhibitors, further stabilizing the closed inactive conformation of SHP2 [29]. SHP244 and site 1 inhibitors such as RMC-4550 may cooperatively lock the inactive state of SHP2, delaying its transformation to the open conformation driven by FGFR feedback activation, as suggested by the effective ERK inhibition in the combination treatment group but neither of the single agent treatment group in both JHH-7 and Hep3B cells (Figure 3F). Similarly, disrupting the interaction between RAS and SOS1 abolished the effects of the accumulated pool of open conformation SHP2, as judged by the delay of RMC-4550-induced p-ERK rebound by BAY-293 co-treatment (Supplementary Figure 2G).

Consistent with this notion that FGFR feedback activation drives SHP2 conformational activation, allosteric SHP2 inhibitors are not effective against several disease-associated SHP2 mutants such as E76K that disrupt the auto-inhibition binding interface and cause SHP2 to maintain in an open conformation [38, 39]. We also confirmed that expression of SHP2^E76K^ rendered up to 100 μM SHP099 ineffective inhibiting ERK activity (Supplementary Figure 6B). We further demonstrated that hyper-activation of EGFR through high-dose EGF stimulation rendered SHP394 ineffective against wild-type SHP2 as judged by the lack of inhibition of EGF-induced ERK phosphorylation in Detroit 562 (Supplementary Figure 6C). Presumably, the equilibrium of SHP2 conformation may shift toward the open conformation and higher dosage of SHP2 inhibitors is required to be effective in this scenario. Also under this hypothesis, similar to ATP-competitive FGFR inhibitors, SHP2 active site inhibitors may be less affected by the accumulation of active conformation SHP2 and more effective inhibiting FGFR-activated MAPK signaling. However, we failed to validate any commercially available active site inhibitors of SHP2 including NSC-87877 [40] and IIB-08 [41] to enable such testing (Supplementary Figure 6D), which was also noted by a recent report [42]. We also performed a co-immunoprecipitation assay of SHP2 to examine the binding of other members within the adaptor complex such as FRS2, GAB1, and GRB2 as a readout of SHP2 conformational change following 15-minute or two-hour treatment with SHP2 inhibitors. However, we could not detect the interaction between SHP2 and its interactors at the baseline level with robust SHP2 immunoprecipitation in these models (data not shown).

Using a previously reported selumetinib-induced phospho-MEK assay [32], we demonstrated that the rapid feedback activation of FGFRs was a result of ERK inhibition rather than a unique feature of allosteric inhibitor binding to SHP2. Interestingly, there was a strong induction of p-SHP2 (Y542) in all FGFR-driven cell lines following selumetinib treatment, which was not observed in the EGFR-driven Detroit 562 cells (Figure 4A; Supplementary Figure 3A). Y542 site of SHP2 undergoes phosphorylation in response to upstream RTK activation and p-Y542 has been suggested to promote the relief of SHP2 from auto-inhibition and GRB2/SOS recruitment to SHP2 [9, 31]. Phospho-SHP2 levels were also elevated following two-hour RMC-4550 treatment in JHH-7 and Hep3B cells (Supplementary Figure 2H). We also found that FGFR-driven feedback activation was more resistant to SHP2 inhibition compared with EGFR-driven feedback activation in an isogenic model NCI-H522 (Figure 4B). Consistent with these observations, p-SHP2 induction was only observed when FGFR was driving the feedback activation (Figure 4B). These data further support our hypothesis that feedback activated FGFRs efficiently promote SHP2 activation, possibility leading to resistance to allosteric inhibitors. We have previously proposed that induction of p-SHP2 can be a biomarker to identify SHP2 dependent feedback activation in KRAS mutant cell lines treated with MEK inhibitors [32] and the new insights from this study suggest that substantially high p-SHP2 induction might be associated with resistance to allosteric SHP2 inhibitors. Importantly, this resistance to SHP2 inhibition may also occur in KRAS or BRAF mutant cancers wherein FGFRs are feedback activated due to MAPK inhibition, as evidenced by the observations in SW1736 cells (Supplementary Figure 6A). Therefore, the combination of SHP2 and MAPK inhibitors for treating KRAS or BRAF mutant cancers may not benefit all patients when FGFRs are involved in the feedback activation.

It is also worth pointing out that allosteric SHP2 inhibitors can effectively inhibit FGFR-activated SHP2 such as in SNU-398 cells for both growth and pathway inhibition (Supplementary Figure 6E). We also previously reported a KRAS mutant cancer cell line HUP-T3 with FGFR-driven feedback activation following selumetinib treatment, which can be inhibited by 5 μM SHP099 [32]. The underlying mechanism of the varying sensitivity of FGFR-activated SHP2 to allosteric inhibitors could be related to cellular context and the feedback activation of parallel signaling pathways and requires further investigation. This information will be helpful to guide the patient selection strategy for the clinical development of SHP2 inhibitors not only in cancers with genetic alterations of FGFR such as gastric cancer (*FGFR2* amplification) [43], hepatocellular carcinoma (FGF19-FGFR4 activation) [44] and cholangiocarcinoma (FGF19-FGFR4 activation) [45] but also in KRAS or BRAF mutant cancers in combination with MAPK inhibitors to prevent the pathway feedback activation. Conceivably, FGFR activation may also emerge as an adaptive resistance mechanism to SHP2 inhibitors in pre-clinical models and cancer patients treated with SHP2 inhibitors such as RMC-4630 (ClinicalTrials. gov Identifier: NCT03634982), JAB-3068 (NCT03518554), and JAB-3312 (NCT04045496). Our findings that certain RTKs such as FGFRs are resistant to SHP2 inhibitors may also raise the possibility that SHP2 inhibitors might be better tolerated than pan-RTK inhibitors and provide a possible explanation of differences between SHP2 and FGFR inhibitors observed in pre-clinical and clinical studies. Moreover, despite the resistance in FGFR-driven cells, SHP2 inhibitors may prevent feedback activation of other RTKs such as ERBB after prolonged FGFR inhibition as evidenced by the *in vitro* combination synergy (Figure 6B, 6C). These data support SHP2 inhibitor as a novel combination partner with FGFR inhibitors for treating FGFR-driven cancers in the clinic in addition to combination with MEK inhibitors as previously reported [46]. Lastly, our findings may provide a valuable system to identify next generation SHP2 inhibitors that will be more effective inhibiting feedback activation of SHP2 by RTKs.

## MATERIALS AND METHODS

### Cell lines and drugs

Human cancer cell lines originated from the CCLE [18] were authenticated by single-nucleotide polymorphism analysis and tested for mycoplasma infection using a PCR-based detection technology (RADIL, University of Missouri, Columbia, MO; now IDEXX Laboratories). All cell lines used were directly thawed from the CCLE collection stock and cultured as previously described [18] except SW1736 (Creative Bioarray). Cell lines were used within 15 passages of thawing and continuously cultured for less than 6 months. All small molecule inhibitors used were synthesized and structurally verified by NMR and LC/MS according to cited references at Novartis except RMC-4550 (Selleck Chemicals), NSC-87877 (Selleck Chemicals), and IIB-08 (Millipore Sigma). EGF and FGF19 were purchased from R&D Systems.

### PTPN11 knockdown using siRNA or shRNA

The siRNA SMARTpool targeting SHP2 or FRS2 were purchased from Dharmacon (#L-003947-00-0005 and #L-006440-00-0005) and transfected into JHH-7 and Hep3B cells using DharmaFECT 1 Transfection Reagent (Dharmacon # T-2001-02) following the manufacturer’s instruction. Three days after transfection, cell lysates were collected for immunoblot analysis. Doxycycline-inducible shRNA targeting SHP2 was introduced into JHH-7 and Hep3B cells with retrovirus packaged using the pRSIUP-U6TetSHP2-shRNA-UbiC-TetRep-2A plasmid [47] and selection by 1 μg/mL puromycin in tetracycline (TET)-free media (Clonetech). Cell lysates were collected for analysis after 5-day treatment of 100 ng/ml doxycycline.

### Over-expression of SHP2 and SPRY4

Doxycycline-inducible shRNA-resistant wild-type SHP2 or SHP2^E76K^ cDNA was introduced into cells with retrovirus packaged using the pLKO-SHP2 plasmid [10] and selection by 1 mg/ml geneticin in TET-free media (Clonetech). Doxycycline-inducible SPRY4 cDNA was introduced into JHH-7 and Hep3B cells with retrovirus packaged using the Retro-X Tet-On 3G System (Clonetech) and selected by 1 μg/mL puromycin in TET-free media (Clonetech). Cells were treated with 100 ng/ml doxycycline as indicated to induce SPRY4 over-expression.

### qRT-PCR

qRT-PCR was performed and analyzed as reported [29]. Taqman gene expression probes for *PTPN11* (Hs01590340_gH), *FRS2* (Hs01552856_m1), *SPRY1* (Hs01083036_s1), *SPRY2* (Hs01921749_s1), *SPRY3* (Hs00538856_m1), and *SPRY4* (Hs01935412_s1) were purchased from ThermoFisher. Human β-actin (Applied Biosystems; #4310881E) was used as an internal control.

### RAS-GTP assay, ELISA, and immunoblotting

About half million cells per well were cultured in 6-well plates overnight before treated with SHP394 or BGJ398 for 15 minutes or two hours. RAS-GTP levels were measured by the G-LISA RAS activation assay kit (Cytoskeleton #BK-131) following the manufacturer’s instruction. Phospho-FGFR4 levels were measured by the Phospho-FGF Receptor 4 (panTyr) Sandwich ELISA Kit (CST #69193) following the manufacturer’s instruction. Immunoblotting was performed as previously described [10] and tubulin served as a loading control in all immunoblot experiments. Signals for p-ERK, ERK, p-AKT, and AKT bands were quantified using Odyssey LI-COR software and the p-ERK/ERK and p-AKT/AKT signal ratios were calculated and normalized to DMSO. The following antibodies were used: p-MEK (CST #9154), p-ERK (CST #4370), ERK (CST #4695), p-RSK3 (CST #9348), p-AKT (CST #4060), AKT (CST #9272), FGFR4 (CST #8562), p-EGFR (CST #3777), p-FRS2 (Y436, CST #3861), p-SHP2 (Abcam #ab62322), SHP2 (CST #3397), SPRY4 (R&D System #AF5070), and Tubulin (CST #3873).

### Phospho-ERK MSD assay

Twenty thousand cells per well were cultured in 96-well plates overnight and treated with SHP099 at 3 fold serial dilutions from 100 μM for 15 minutes or two hours. Cells were lysed with 50 μl complete lysis buffer in the MSD phospho-ERK assay kit (Meso Scale Discovery # K151DWD-1) and processed according to the manufacturer’s instruction. Protein concentrations were quantified using the BCA assay as previously reported [32]. Phospho-ERK levels were normalized by the total protein amount and compared with the DMSO control.

### Cell proliferation assay and colony formation assay

The high-throughput compound profiling of SHP099, BGJ398, erlotinib, and trametinib was conducted as previously reported [18]. For individual compound testing, around 2,000 cells were seeded in 96-well plates the day before they were treated with compounds of serial dilutions. After 6 days of treatment, cell viability was measured by the CellTiter-Glo Assay (Promega) according to the manufacturer’s instruction. For colony formation assay, 10,000 cells per well were seeded in 6-well plates the day before they were treated with 100 ng/ml doxycycline and/or compounds. Media with doxycycline or compounds were replaced every 5 days. After 14-21 days, cell colonies were stained with crystal violet as described.

### Tumor xenograft experiments

All animal studies were performed in accordance with the NIH Guide for the Care and Use of Laboratory Animals. Patient-derived hepatocellular carcinoma (HCC) xenograft line HCC09-0913 was established and implanted in male C. B-17 SCID mice aged 9–10 weeks and weighed 23–25 g (InVivos Pte. Ltd., Singapore) as described previously [48]. FGFR4 inhibitor FGF401 [25] was prepared in 100 mM citrate buffer (pH=2.5) and SHP099 was prepared in 0.5% (w/v) hydroxypropyl methylcellulose aqueous solution. Mice were monitored every 2–3 days and tumors were measured and calculated by the following formula: Tumor volume = [(length) × (width^2^) × (π/6)]. Once tumors reached roughly 150–175 mm^3^, mice were randomly assigned to receive either vehicle, SHP099 (100 mg/kg daily), or FGF401 (30 mg/kg twice daily) by oral gavage for 14 days. At the end of the study, mice were euthanized two hours following the last dose and tumor fragments were collected for immunoblotting [48].

## SUPPLEMENTARY MATERIALS




