# Supplementary material for: Resistance to allosteric SHP2 inhibition in FGFR-driven cancers through rapid feedback activation of FGFR
Source: Oncotarget. 2020 Jan 21;11(3):265–81. doi: 10.18632/oncotarget.27435 (PMC6980623; doi:10.18632/oncotarget.27435)
Supplement: Supplementary file 1 [file oncotarget-11-265-s001.pdf]

Resistance to allosteric SHP2 inhibition in FGFR-driven cancers through rapid feedback activation of FGFR

SUPPLEMENTARY MATERIALS

1. McDonald ER 3rd, de Weck A, Schlabach MR, Billy E, Mavrakis KJ, Hoffman GR, Belur D, Castelletti D, Frias E, Gampa K, Golji J, Kao I, Li L, et al. Project DRIVE: A compendium of cancer dependencies and synthetic lethal relationships uncovered by large-scale, deep RNAi screening. Cell. 2017; 170:577–592.e10. https://doi.org/10.1016/j.cell.2017.07.005. [PubMed]

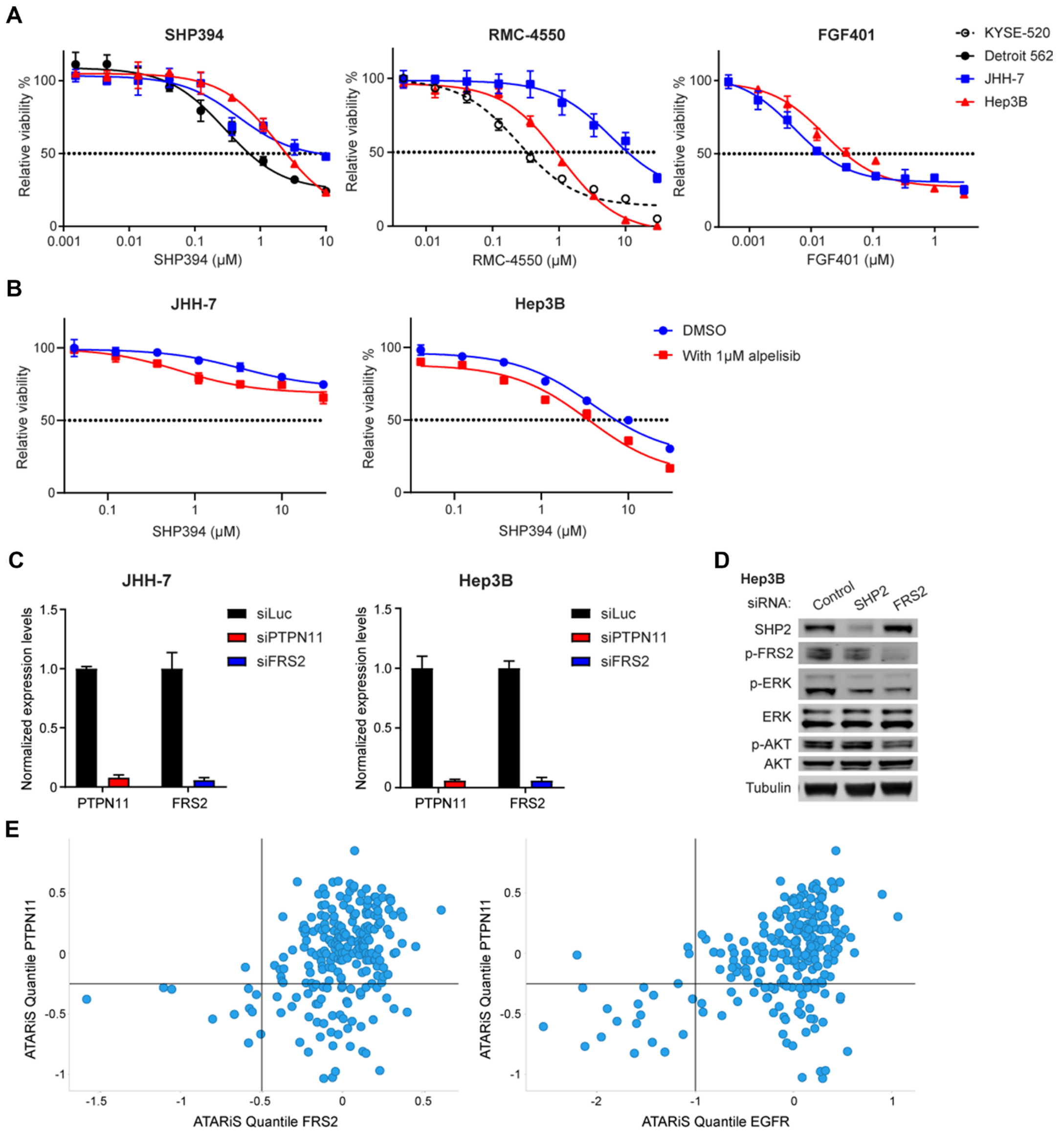

Supplementary Figure 1: (**A**) Anti-proliferative effects of SHP394, RMC-4550, and FGF401 at the indicated concentrations in a 6-day cell proliferation assay with indicated cell lines (mean percentages of cell viability are shown, error bars, SD; $n = 3$). (**B**) Anti-proliferative effects of SHP394 at the indicated concentrations in the absence or the presence of PI3Kα inhibitor alpelisib in a 3-day cell proliferation assay with indicated cell lines (mean percentages of cell viability are shown, error bars, SD; $n = 3$). (**C**) mRNA levels determined by qRT-PCR of *PTPN11* (encoding SHP2) and *FRS2* in JHH-7 cells transfected with scrambled control siRNA or siRNA targeting either *PTPN11* (siPTPN11) or *FRS2* (siFRS2) for 3 days. (**D**) Immunoblot of indicated proteins in Hep3B cells transfected with siRNAs targeting SHP2 or FRS2 or scrambled control siRNA for 2 days. (**E**) Correlation of dependencies on PTPN11 and FRS2 (a surrogate for FGFRs) or EGFR using ATARiS scores from a pooled shRNA screen with 234 CCLE cell lines from Project DRIVE [1]. Each dot represents one cell line.

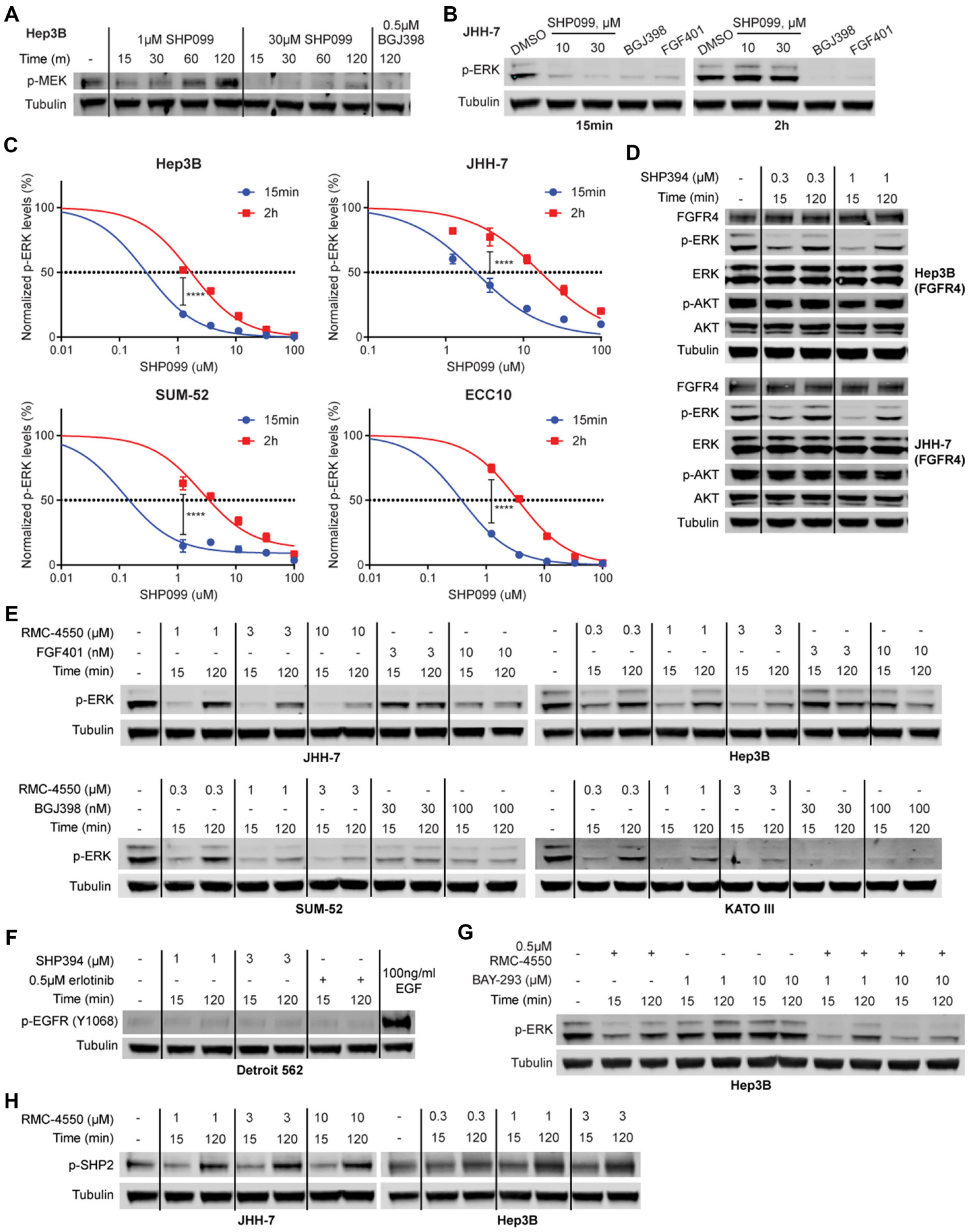

Supplementary Figure 2: (**A**) Immunoblot of p-MEK and tubulin in Hep3B cells treated with 1 µM or 30 µM SHP099 for 15 min, 30 min, 60 min, or 120 min, or 0.5 µM BGJ398 for 2 h. (**B**) Immunoblot of p-ERK and tubulin in JHH-7 cells treated with 10 µM or 30 µM SHP099, 0.5 µM BGJ398, or 0.1 µM FGF401 for 15 min or 2 h. (**C**) Phospho-ERK levels measured by Meso Scale Discovery (MSD) assays of indicated cells treated with SHP099 at indicated concentrations for either 15 min or 120 min. Signals are first normalized to the total protein amount and then to DMSO-treated control (error bars, SD; *n* = 3). ****, $p < 0.0001$ (by *t*-test). (**D**) Immunoblot of indicated proteins in Hep3B and JHH-7 cells treated with 0.3 µM or 1 µM SHP394 for 15 min or 2 h. (**E**) Immunoblot of p-ERK and tubulin in indicated cells treated with either RMC-4550, FGF401, or BGJ398 at indicated concentrations for 15 min or 120 min. (**F**) Immunoblot of p-EGFR (Y1068) and tubulin in Detroit 562 cells treated with SHP394 or erlotinib at indicated concentrations for 15 min or 120 min or 100 ng/ml EGF for 15 min. (**G**) Immunoblot of p-ERK and tubulin in Hep3B cells treated with 0.5 µM RMC-4550, the RAS-SOS1 interaction inhibitor BAY-293 (1 or 10 µM), or in combination for either 15 min or 120 min. (**H**) Immunoblot of p-SHP2 and tubulin in JHH-7 and Hep3B cells treated with RMC-4550 at indicated concentrations for 15 min or 120 min.

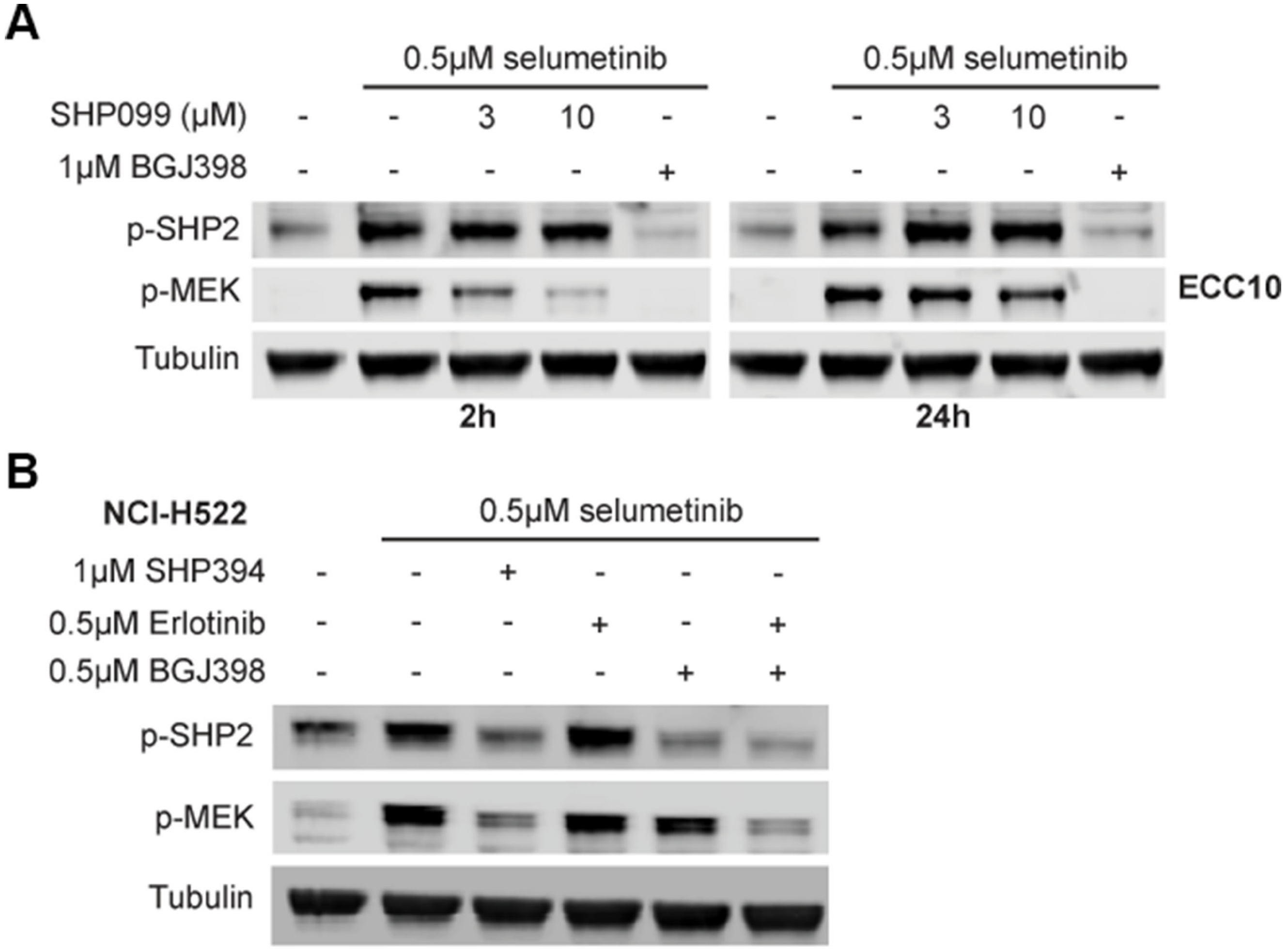

Supplementary Figure 3: (**A**) Immunoblot of p-SHP2, p-MEK, and tubulin in ECC10 cells treated with 0.5 μM selumetinib alone or in combination with 3 or 10 μM SHP099 or 1 μM BGJ398 for either 2 h or 24 h. (**B**) Immunoblot of p-SHP2, p-MEK, and tubulin in NCI-H522 cells treated with 0.5μM selumetinib alone or in combination with 1 μM SHP394, 0.5 μM erlotinib, 0.5 μM BGJ398, or erlotinib plus BGJ398.

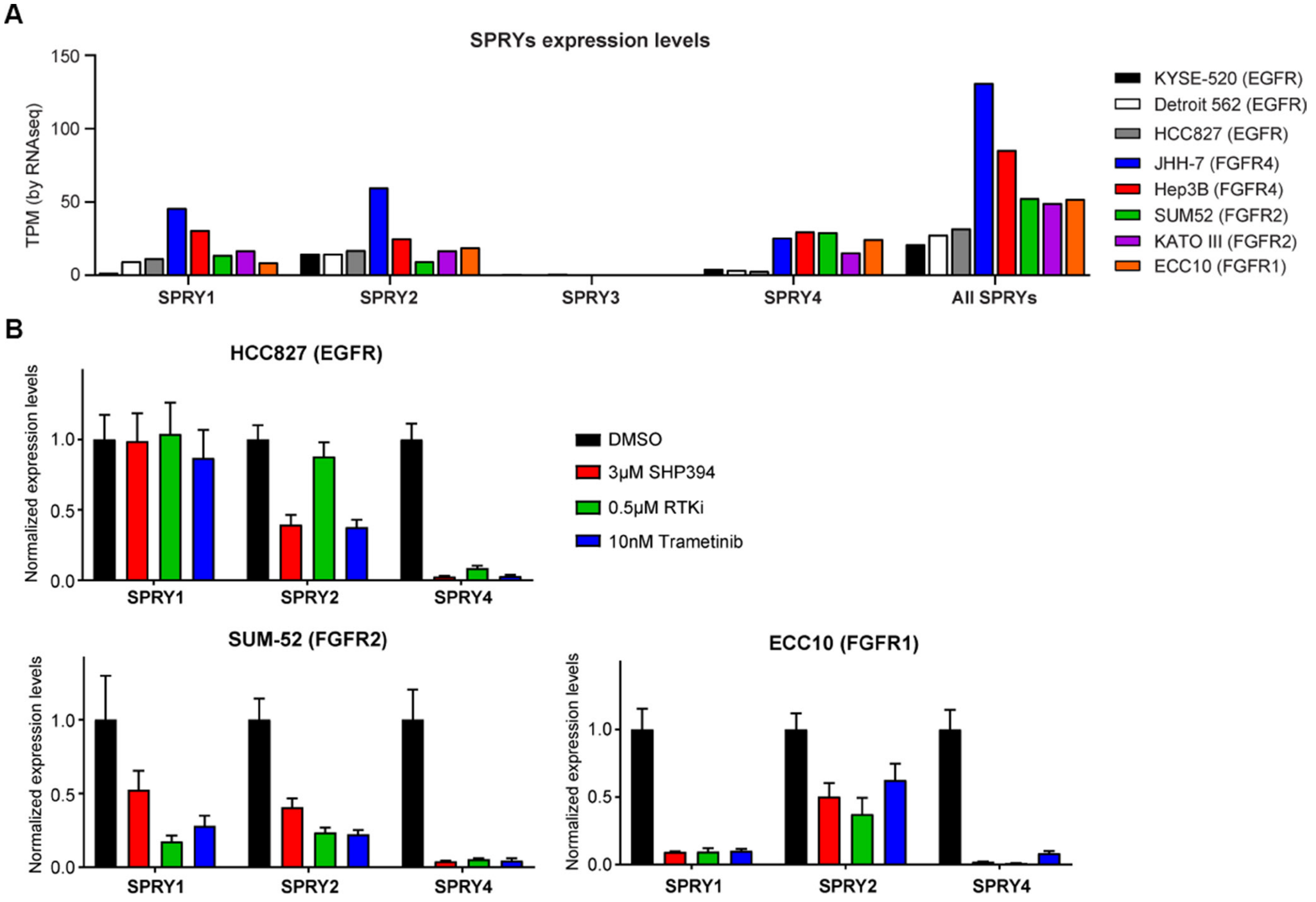

Supplementary Figure 4: (**A**) mRNA levels of *SPRY1*, *SPRY2*, *SPRY3*, and *SPRY4* in indicated cell lines determined by RNAseq (TPM, transcript per million reads). (**B**) mRNA levels determined by qRT-PCR of *SPRY1*, *SPRY2*, and *SPRY4* in HCC827, SUM-52, and ECC10 cells treated with DMSO, 3 μM SHP394, 0.5 μM RTK inhibitor (erlotinib for HCC827 and BGJ398 for SUM-52 and ECC10), or 10 nM trametinib for 2 h.

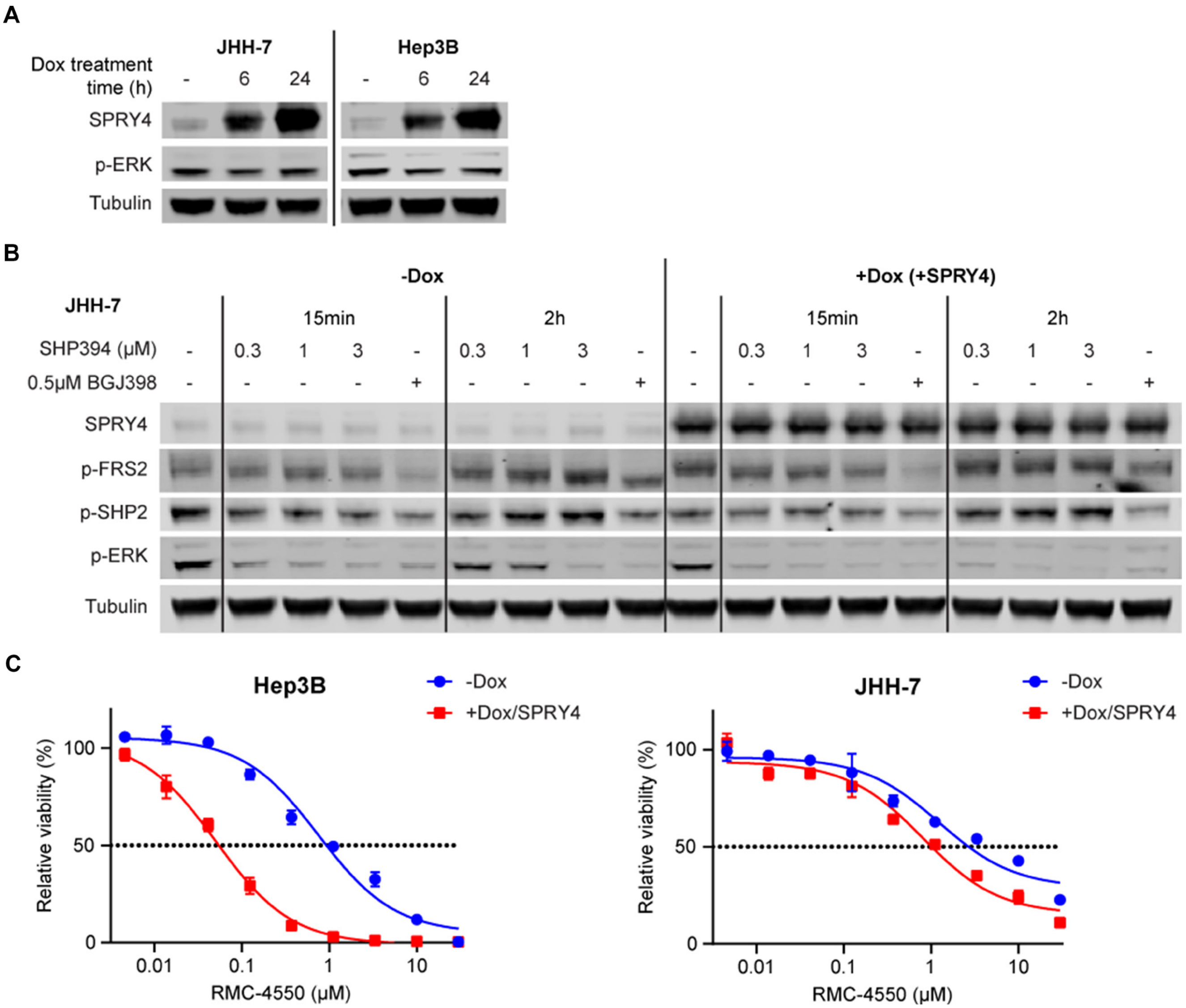

Supplementary Figure 5: (**A**) Immunoblot of SPRY4, p-ERK, and tubulin in JHH-7 and Hep3B cells stably expressing doxycycline-inducible SPRY4 treated with PBS (-) or 100 ng/ml doxycycline for either 6 or 24 h (**B**) Immunoblot of indicated proteins in JHH-7 cells stably expressing doxycycline-inducible SPRY4 treated with PBS (−Dox) or 100 ng/ml doxycycline (+SPRY4) for 48 h followed by treatment with DMSO, SHP394 at indicated concentrations, or 0.5 µM BGJ398 for either 15 min or 2 h. (**C**) Anti-proliferative effects of RMC-4550 at the indicated concentrations in a 6-day cell proliferation assay with Hep3B and JHH-7 cells stably expressing doxycycline-inducible SPRY4 in the absence (−Dox) or presence (+Dox/SPRY4) of 100 ng/ml doxycycline (mean percentages of cell viability are shown, error bars, SD; *n* = 3).

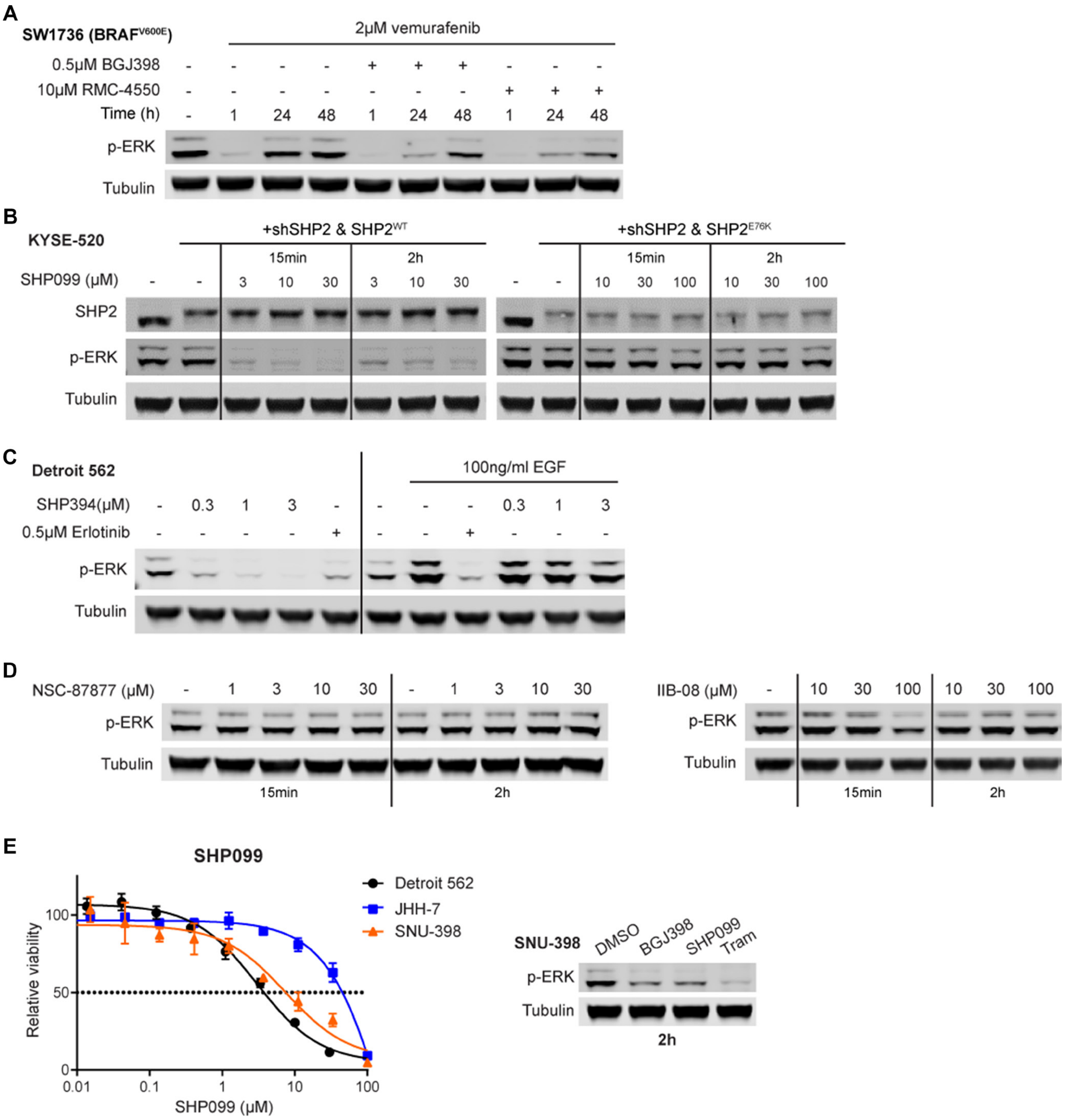

Supplementary Figure 6: (**A**) Immunoblot of p-ERK and tubulin in SW1736 cells treated with 2 µM vemurafenib alone or in combination with either 0.5 µM BGJ398 or 10 µM RMC-4550 for 1 h, 24h, or 48h. (**B**) KYSE-520 cells stably expressing doxycycline-inducible shRNA targeting SHP2 (shSHP2) and doxycycline-inducible shSHP2-resistant wild-type SHP2 or SHP2^{E76K} were treated with PBS (first lane in each blot) or 100 ng/ml doxycycline (+shSHP2 & SHP2^{WT} or +shSHP2 & SHP2^{E76K}). Then doxycycline-treated cells were treated with DMSO or SHP099 at indicated concentrations for either 15 min or 2 h. Cell lysates were collected and SHP2 and p-ERK levels were determined by immunoblotting. (**C**) Detroit 562 cells were first treated with DMSO, SHP394 at indicated concentrations, or 0.5 µM erlotinib for 15 min. Then either PBS or 100 ng/ml EGF was added into medium in each treatment group as indicated and incubated for another 15 min. Cell lysates were collected and p-ERK levels were determined by immunoblotting. (**D**) Immunoblot of p-ERK and tubulin in KYSE-520 cells treated with NSC-87877 or IIB-08 at indicated concentrations respectively for either 15 min or 2 h. (**E**) Anti-proliferative effects of SHP099 at the indicated concentrations in a 6-day cell proliferation assay with SNU-398 (mean percentages of cell viability are shown, error bars, SD; *n* = 3) and immunoblot of p-ERK and tubulin in SNU-398 cells treated with 0.5 µM BGJ398, 10 µM SHP099, or 0.1 µM trametinib for 2 h.

Supplementary Table 1: IC_{50} values for SHP099, erlotinib, BGJ398, and trametinib in 262 MAPK wild-type cancer cell lines.
See Supplementary Table 1

Supplementary Table 2: IC_{50} values for erlotinib, BGJ398, SHP099, and trametinib in indicated cell lines

Cell line	Erlotinib IC_{50} (µM)	SHP099 IC_{50} (µM)	Trametinib IC_{50} (µM)
ncih3255	0.073171362	0.031650614	1.32493508
hcc827	0.032858856	0.207281575	30
hcc4006	0.051653374	1.29051304	0.124661632
pc14	0.068982363	2.24941182	3.89205265
hsc2	1.06147218	2.47644019	0.907467127
ncih2073	0.563343346	4.12305164	0.212492675
te11	1.00000036	5.70292711	0.351163566
cal851	0.836462617	6.629848	0.268465161
snu1079	0.485541403	8.74079418	0.206499666
corl105	0.390687704	9.33290386	0.07003773

Cell line	BGJ398 IC_{50} (µM)	SHP099 IC_{50} (µM)	Trametinib IC_{50} (µM)
1321n1	0.06153952	13.7569447	1.13786888
g401	0.27465263	20.9907684	0.094869465
a172	0.072773717	23.3831024	0.268476963
snu878	1.00416183	24.7266006	0.176364928
hep3b217	0.132478997	30	0.019987924
msto211h	0.073684432	30	0.077734217
snu398	0.847619236	30	0.058884621
ncih716	0.00801891	30	0.04852856
mfe296	0.630353451	30	0.418355763
mfm223	0.054296296	30	0.541348934
katoiii	0.023155097	30	0.374544024
59m	0.508683026	30	30
jhuem2	0.098074257	30	0.450424314
snu16	0.027185481	30	0.904276073
jhh7	0.236970827	30	0.011795435
hep3b	0.099752091	30	0.023189209
sum52pe	0.007300598	30	0.080476783

Supplementary Table 3: Statistical analysis for Figure 5A by paired *t*-test

Cell lines	DMSO vs. 3 µM SHP394	DMSO vs. 0.5 µM BGJ398	DMSO vs. 10 nM trametinib
Hep3B	**, $p = 0.0023$	***, $p = 0.0004$	***, $p = 0.0003$
JHH-7	**, $p = 0.0073$	**, $p = 0.0016$	**, $p = 0.0027$
Detroit 562	n. s. ($p = 0.0694$)	n. s. ($p = 0.1627$)	n. s. ($p = 0.1065$)
KYSE-520	n. s. ($p = 0.0727$)	n. s. ($p = 0.0728$)	n. s. ($p = 0.1067$)

n. s., not significant.
