# Supplementary material for: Resistance to allosteric SHP2 inhibition in FGFR-driven cancers through rapid feedback activation of FGFR
Source: Oncotarget. 2020 Jan 21;11(3):265–81. doi: 10.18632/oncotarget.27435 (PMC6980623; doi:10.18632/oncotarget.27435)
Supplement: Supplementary file 2 [file oncotarget-11-265-s002.docx]

**Supplementary Table 1: IC_50_ values for SHP099, erlotinib, BGJ398, and trametinib in 262 MAPK wild-type cancer cell lines**

| Cell line | SHP099 IC_50_ (µM) | Erlotinib IC_50_ (µM) | BGJ398 IC_50_ (µM) | Trametinib IC_50_ (µM) |
| --- | --- | --- | --- | --- |
| eol1 | 0.027610494 | 1.9077754 | 3.85511351 | 0.005041324 |
| ncih3255 | 0.031650614 | 0.073171362 | 9.69088554 | 1.32493508 |
| hcc1143 | 0.067369066 | 15.7699213 | 30 | 0.536052048 |
| hcc827 | 0.207281575 | 0.032858856 | 30 | 30 |
| bt16 | 0.753495216 | 1.65417922 | 8.30815315 | 0.189470038 |
| mv411 | 1.02877355 | 3.36227918 | 4.59335136 | 0.017293053 |
| hcc4006 | 1.29051304 | 0.051653374 | 7.10014629 | 0.124661632 |
| kbm7 | 1.3071512 | 5.71220779 | 3.12694836 | 0.011438359 |
| uke1 | 1.50356579 | 9.31548882 | 30 | 0.003174256 |
| sknbe2 | 1.78172171 | 30 | 5.81082439 | 0.048845217 |
| pc14 | 2.24941182 | 0.068982363 | 14.0347881 | 3.89205265 |
| hsc2 | 2.47644019 | 1.06147218 | 14.1882715 | 0.907467127 |
| ocim1 | 2.51847458 | 15.8892393 | 3.07248902 |  |
| ociaml5 | 2.98530436 | 11.0623846 | 30 | 0.012271848 |
| ncih2073 | 4.12305164 | 0.563343346 | 9.60587311 | 0.212492675 |
| gdm1 | 4.20434093 | 8.46725368 | 1.6106497 | 0.09718401 |
| monomac1 | 4.67266369 | 3.57783723 | 3.07295632 | 0.412258893 |
| te11 | 5.70292711 | 1.00000036 | 3.56637764 | 0.351163566 |
| bicr31 | 6.08269644 | 1.62536669 | 13.5365534 | 0.457965851 |
| ccrfhsb2 | 6.51317978 | 7.66351843 | 1.43753064 | 6.21059322 |
| cal851 | 6.629848 | 0.836462617 | 30 | 0.268465161 |
| igrov1 | 6.89141321 | 1.63105512 | 30 | 0.101773448 |
| te617t | 7.30155182 | 1.41232693 | 30 | 0.174457073 |
| lc1sqsf | 7.34177113 | 26.224102 | 14.8929501 | 2.05639267 |
| pecapj49 | 7.35614252 | 1.35216951 | 12.7145748 | 0.552676499 |
| fu97 | 8.54665852 | 13.7623873 | 0.814104915 | 1.13822532 |
| snu1079 | 8.74079418 | 0.485541403 | 30 | 0.206499666 |
| achn | 9.09702778 | 1.14786339 | 30 | 30 |
| corl105 | 9.33290386 | 0.390687704 | 30 | 0.07003773 |
| bhy | 9.41074371 | 1.50459027 | 8.35892773 | 0.367585421 |
| mkn45 | 9.67939377 | 16.0750084 | 5.655756 | 0.023222864 |
| ks1 | 9.73206902 | 8.59377384 | 30 | 0.129653916 |
| mfe319 | 10.1666088 | 3.88888955 | 12.9645271 | 0.244599462 |
| cal54 | 10.4306755 | 1.57270336 | 4.26486206 | 1.54407752 |
| hsc3 | 10.4403067 | 1.49605203 | 6.49160528 | 0.087241076 |
| kasumi6 | 10.5098171 | 3.52791786 | 4.00808859 | 1.15475571 |
| sw48 | 10.5322409 | 7.8497963 | 4.63146925 | 0.002550051 |
| hsc4 | 11.335762 | 1.47499871 | 12.1193581 | 0.214283764 |
| 769p | 11.4104815 | 0.785359502 | 9.32470417 | 0.904858828 |
| ebc1 | 11.5291843 | 30 |  | 0.032361586 |
| caki1 | 12.0275688 | 0.668308973 | 30 | 30 |
| bicr16 | 12.3790798 | 2.11765456 | 6.98578501 | 0.156000406 |
| ncih2110 | 12.985054 | 4.61176872 |  | 1.96772039 |
| ncih1650 | 13.0436764 | 2.13531661 | 30 | 3.15700173 |
| bicr6 | 13.0666246 | 5.38921309 | 30 | 0.078615397 |
| 1321n1 | 13.7569447 | 21.8280201 | 0.06153952 | 1.13786888 |
| cal29 | 13.9812317 | 7.74817753 | 30 | 5.44767046 |
| lc1f | 15.7380896 | 22.4863567 | 30 | 30 |
| cov644 | 15.9427462 | 2.67793441 | 3.83896804 | 0.27577135 |
| detroit562 | 16.7496452 | 8.04820251 | 6.73438549 | 12.1738052 |
| scc25 | 17.3837051 | 2.08556128 | 14.4614744 |  |
| hcc1806 | 18.8564491 | 6.01530981 | 30 | 30 |
| g401 | 20.9907684 | 12.383976 | 0.27465263 | 0.094869465 |
| me180 | 21.4981594 | 3.40061784 | 3.92209983 | 1.0173316 |
| ncih1568 | 21.8347359 | 2.54713559 | 2.88883185 | 0.390953928 |
| monomac6 | 22.77911 | 6.0296011 | 30 | 0.193046466 |
| a172 | 23.3831024 |  | 0.072773717 | 0.268476963 |
| hcc95 | 23.4917641 | 30 | 30 | 30 |
| cl14 | 23.7951202 | 7.21290827 | 3.75887632 | 0.231639594 |
| snu878 | 24.7266006 | 4.3553977 | 1.00416183 | 0.176364928 |
| snu761 | 25.5290585 | 10.6957254 | 8.71203232 | 0.025957849 |
| bc3c | 29.940403 | 2.03450513 | 1.61973357 | 7.88887453 |
| ncih196 | 30 | 30 | 30 | 30 |
| bxpc3 | 30 | 4.07916689 | 8.53918648 | 0.031277284 |
| hep3b217 | 30 | 30 | 0.132478997 | 0.019987924 |
| snu324 | 30 | 6.36313438 | 6.58816481 | 0.306203842 |
| u937 | 30 | 12.994895 | 30 | 0.121683709 |
| kmrc20 | 30 | 30 | 30 | 30 |
| istmes2 | 30 | 6.53252888 | 1.77937424 | 6.92115641 |
| ovise | 30 | 10.7880125 | 30 | 1.35872209 |
| sem | 30 | 8.76868725 | 4.00323248 | 0.024582803 |
| sr786 | 30 | 30 |  | 0.584133923 |
| no36 | 30 | 30 | 30 | 30 |
| ncih1915 | 30 | 12.6268749 | 11.1399527 | 0.016887877 |
| li7 | 30 | 17.8540497 | 3.75908494 | 0.192732632 |
| scaber | 30 | 3.00762439 | 30 | 30 |
| ncih2286 | 30 | 9.93997478 | 6.69205046 | 1.60714817 |
| hcc78 | 30 | 30 | 16.4451351 | 4.25409889 |
| ncih2171 | 30 | 30 | 30 |  |
| pc3 | 30 | 24.800333 | 30 | 0.612962425 |
| hara | 30 | 12.8606834 | 9.77128124 | 0.476075679 |
| hlf | 30 | 29.1049347 | 9.13508606 | 0.574157834 |
| a498 | 30 | 13.3798256 | 5.71710491 | 5.45329905 |
| kns62 | 30 | 30 | 30 | 0.106383093 |
| cas1 | 30 | 30 | 12.2966909 | 0.395403117 |
| mdamb453 | 30 | 7.84651995 | 2.11857677 | 0.185547844 |
| msto211h | 30 | 10.9682322 | 0.073684432 | 0.077734217 |
| kuramochi | 30 | 30 | 6.51632881 | 0.473738223 |
| db | 30 | 7.63477802 | 4.98119783 | 0.589299023 |
| ncih2452 | 30 | 30 | 30 | 30 |
| cmk | 30 | 15.2077646 | 30 | 30 |
| gi1 | 30 | 19.6944981 | 8.01379204 | 0.345879614 |
| lmsu | 30 | 8.51295853 | 1.18717504 | 0.450128734 |
| hel9217 | 30 | 7.46901321 | 30 | 1.13038862 |
| gamg | 30 | 30 | 30 | 30 |
| ln229 | 30 | 14.0820503 | 7.55683517 | 0.322338313 |
| snu387 | 30 | 30 | 6.51074743 | 1.92656338 |
| rpmi8402 | 30 | 12.2747993 | 2.94057846 | 0.045838889 |
| rdes | 30 | 8.54485226 | 3.16074991 | 30 |
| ncih1703 | 30 | 16.4755154 | 30 | 1.05144405 |
| j82 | 30 | 30 | 30 | 30 |
| ncih661 | 30 | 30 | 17.2228794 | 1.56125689 |
| ncih520 | 30 | 30 | 1.29648161 | 1.70440352 |
| kns60 | 30 | 30 | 30 | 30 |
| snu398 | 30 | 30 | 0.847619236 | 0.058884621 |
| daoy | 30 | 30 | 30 | 4.66671228 |
| rerflcms | 30 | 15.7380896 | 17.4890633 | 2.1172359 |
| u118mg | 30 | 19.9002552 | 30 | 30 |
| jhh4 | 30 | 5.77036333 | 2.43500066 | 0.118830986 |
| ncih716 | 30 | 18.5378532 | 0.00801891 | 0.04852856 |
| kyse30 | 30 | 30 | 30 | 0.071411446 |
| ncih841 | 30 | 8.31806946 | 12.1441498 | 0.636029959 |
| kyse70 | 30 | 15.7456989 | 30 | 1.08775139 |
| hel | 30 | 10.8048553 | 4.87465382 | 30 |
| snu886 | 30 | 30 | 30 | 0.047814108 |
| kle | 30 | 30 | 30 | 30 |
| snu685 | 30 | 23.2350864 | 30 | 0.895447969 |
| hcc1500 | 30 | 30 | 3.93452954 | 8.36958694 |
| bt549 | 30 | 14.5232515 | 30 | 2.99774551 |
| ju77 | 30 | 19.2542 | 3.33791399 | 1.05477619 |
| jimt1 | 30 | 29.5054474 | 30 | 0.792571187 |
| snu475 | 30 | 22.6966743 | 14.4405174 | 1.07816184 |
| hec265 | 30 | 30 | 10.101366 | 7.47490454 |
| sw1783 | 30 | 30 | 30 | 30 |
| wm2664 | 30 | 30 | 30 | 0.003316279 |
| vmcub1 | 30 | 29.7240887 | 15.8157425 | 30 |
| skes1 | 30 | 3.24462891 | 30 | 30 |
| sudhl10 | 30 | 13.3425407 | 5.34311056 | 0.436076224 |
| ociaml2 | 30 | 18.9015198 | 30 | 0.000335454 |
| sudhl5 | 30 | 9.79277515 | 5.18568945 | 0.41690588 |
| ncih2170 | 30 | 3.01590633 | 30 |  |
| 921 | 30 | 24.7684498 | 30 | 0.013085316 |
| snu349 | 30 | 30 | 3.12944484 | 1.13084006 |
| sw1088 | 30 | 17.725338 | 30 | 4.27255344 |
| a704 | 30 | 27.6213837 | 4.30572748 | 6.24329424 |
| ncih211 | 30 | 30 | 3.06392169 | 0.15132755 |
| tanoue | 30 | 23.7033138 | 5.83257055 |  |
| mfe296 | 30 | 8.24509907 | 0.630353451 | 0.418355763 |
| mdamb134vi | 30 | 30 | 30 | 0.845127165 |
| efm19 | 30 | 30 | 30 | 30 |
| jl1 | 30 | 30 | 9.03150177 | 0.107447289 |
| mfm223 | 30 | 14.523571 | 0.054296296 | 0.541348934 |
| accmeso1 | 30 | 30 | 13.0227699 | 0.573257923 |
| dms273 | 30 | 30 | 12.6897345 | 1.29633927 |
| molt16 | 30 | 6.54402208 | 2.4725256 | 0.445470363 |
| ncih1341 | 30 | 14.6463118 | 7.10951281 | 4.47235584 |
| ncih810 | 30 | 30 | 7.01548958 |  |
| lk2 | 30 | 19.5251617 | 30 | 0.574885726 |
| ludlu1 | 30 | 30 | 30 | 30 |
| bftc909 | 30 | 30 | 30 | 30 |
| ncih2126 | 30 | 6.50874472 | 30 | 1.13469803 |
| an3ca | 30 | 14.1450033 | 2.7783308 | 14.8710403 |
| bt474 | 30 | 8.67158222 | 10.0651407 | 4.70336008 |
| p3hr1 | 30 | 28.6223068 | 4.19736767 |  |
| karpas422 | 30 | 30 | 4.84912157 | 1.04957509 |
| kms11 | 30 | 28.0396538 | 1.87095296 | 0.414521784 |
| colo320 | 30 | 7.48856878 | 2.8156352 | 4.36841965 |
| igr39 | 30 | 30 | 13.7225275 | 1.53504539 |
| snu423 | 30 | 25.7272778 | 30 |  |
| hcc1954 | 30 | 2.8308959 | 4.79276752 | 1.12392867 |
| mcc142 | 30 | 14.3711958 | 7.0845623 | 0.419110239 |
| kmrc1 | 30 | 21.4109459 | 30 | 5.96830797 |
| sudhl4 | 30 | 20.5720539 | 4.82276917 | 0.563873947 |
| te14 | 30 | 3.78527665 | 30 | 0.8063429 |
| sf268 | 30 | 30 | 30 |  |
| cal51 | 30 | 16.73493 | 5.9340477 | 0.442553282 |
| messa | 30 | 16.5488052 | 3.04579806 | 0.800341308 |
| rmugs | 30 | 7.06621695 | 30 | 0.155220821 |
| caki2 | 30 | 1.30365181 | 30 | 0.259675354 |
| chagok1 | 30 | 30 | 30 | 0.441203684 |
| vmrcrcw | 30 | 19.4527912 | 3.71507192 | 8.72486782 |
| t47d | 30 | 20.4050331 | 30 | 1.67333412 |
| nihovcar3 | 30 | 6.18947697 | 13.3991013 | 0.64844799 |
| cov362 | 30 | 30 | 16.7224312 | 30 |
| nugc3 | 30 | 2.49446249 | 30 | 30 |
| ben | 30 | 30 | 30 | 30 |
| hcc1187 | 30 | 16.7570076 | 30 | 2.18795443 |
| rh41 | 30 | 16.6410599 | 1.21270883 | 0.377290547 |
| mkn1 | 30 | 16.8005104 | 13.2773228 | 14.0585537 |
| corl47 | 30 | 30 | 2.98038888 | 30 |
| katoiii | 30 | 5.42640495 | 0.023155097 | 0.374544024 |
| ncih838 | 30 | 30 | 30 | 0.899763465 |
| ncih2023 | 30 | 27.2123432 | 10.4125853 | 4.05361271 |
| ovcar4 | 30 | 30 | 12.7274323 | 4.86555815 |
| snu449 | 30 | 30 | 30 | 0.511555254 |
| oncodg1 | 30 | 3.38496232 | 5.96581697 | 0.214607552 |
| 59m | 30 | 7.21955109 | 0.508683026 | 30 |
| supt1 | 30 | 16.0083885 | 30 | 30 |
| kns42 | 30 | 20.1811943 |  | 6.22018814 |
| cal148 | 30 | 30 | 30 | 30 |
| mdamb468 | 30 | 3.58949113 |  | 2.72805905 |
| jhuem2 | 30 | 14.0529966 | 0.098074257 | 0.450424314 |
| ncih1437 | 30 | 30 | 30 | 2.35931635 |
| cama1 | 30 | 30 | 12.0016975 | 6.24796009 |
| jhom1 | 30 | 30 | 30 | 30 |
| u266b1 | 30 | 17.9638386 | 4.30932426 | 0.176326185 |
| snu16 | 30 | 30 | 0.027185481 | 0.904276073 |
| jvm2 | 30 | 4.8716464 | 30 | 0.61249727 |
| ncih2052 | 30 | 18.8722 | 30 | 30 |
| sum159pt | 30 | 30 | 30 | 0.674361706 |
| lounh91 | 30 | 8.40220165 | 6.61529636 | 30 |
| 786o | 30 | 22.661293 | 30 | 30 |
| ln18 | 30 | 16.2690239 | 6.30852032 | 5.76136827 |
| ncih2172 | 30 | 30 | 14.3708801 | 14.099699 |
| ncih2228 | 30 | 21.584856 | 30 | 0.527290046 |
| bicr18 | 30 | 30 | 30 | 0.380663067 |
| sw1353 | 30 | 30 | 30 | 30 |
| lo68 | 30 | 30 | 30 | 30 |
| hcc70 | 30 | 9.25103188 | 4.49600315 | 1.42589092 |
| ke97 | 30 | 4.10388994 | 30 | 1.15472054 |
| l428 | 30 | 8.91497707 | 30 | 30 |
| lp1 | 30 | 30 | 5.96675205 | 0.68384701 |
| ncih146 | 30 | 30 | 25.6448765 | 30 |
| cal120 | 30 | 30 | 30 | 2.09159636 |
| mg63 | 30 | 26.9896317 | 4.66414976 | 0.378195256 |
| jhh7 | 30 | 30 | 0.236970827 | 0.011795435 |
| mdamb436 | 30 | 17.870533 | 9.85321045 | 11.1233196 |
| mfe280 | 30 | 11.3856869 | 1.62998033 | 0.883530378 |
| te6 | 30 | 8.13157082 | 30 | 2.20087957 |
| mcc26 | 30 | 30 | 30 | 0.812613785 |
| h4 | 30 | 5.92883444 | 4.6450243 | 0.509223759 |
| sbc5 | 30 | 30 | 30 |  |
| au565 | 30 |  | 30 |  |
| loucy | 30 |  | 30 |  |
| ncin87 | 30 |  | 9.41900253 |  |
| molm16 | 30 |  | 1.60850513 | 0.108646192 |
| zr751 | 30 |  | 15.6899557 | 3.64233875 |
| ncih1975 | 30 |  | 30 | 30 |
| ncih28 | 30 |  | 30 | 11.2912636 |
| sum185pe | 30 |  | 0.050561875 |  |
| snuc1 | 30 |  | 9.31465816 | 0.000852889 |
| ishikawa | 30 | 5.31746054 | 3.90404367 | 0.8265782 |
| mcc13 | 30 | 30 | 30 | 1.29241431 |
| kcimoh1 | 30 | 15.5918036 | 4.85530519 | 0.033129998 |
| sw982 | 30 | 5.88393068 | 30 | 0.697428524 |
| colo699 | 30 | 16.9941559 | 3.75602984 | 0.855816185 |
| cmk86 | 30 | 6.05802059 | 30 | 0.026399609 |
| hep3b | 30 | 4.51292801 | 0.099752091 | 0.023189209 |
| hcc1359 | 30 | 30 | 11.7390556 | 30 |
| kp1n | 30 | 30 | 30 | 30 |
| skmel2 | 30 | 11.8857002 | 4.32801819 | 0.0034428 |
| hrt18 | 30 | 4.292974 | 30 | 0.524631977 |
| kp1nl | 30 | 30 | 30 | 30 |
| sum52pe | 30 | 7.95030212 | 0.007300598 | 0.080476783 |
| colo704 | 30 | 30 | 13.9343071 | 30 |
| vmrclcd | 30 | 30 | 9.56480217 | 5.64842892 |
| hcc1833 | 30 | 30 | 30 | 30 |
| hlc1 | 30 | 11.8606558 | 30 | 0.024394473 |
| wsuwm | 30 | 30 | 4.78855658 |  |
| hle | 30 | 30 | 30 | 0.960416436 |
| snb19 | 30 | 30 | 30 | 30 |
| syo1 | 30 | 23.5947094 | 30 | 0.935986161 |
| colo205 | 30 | 24.5806141 | 30 | 0.006227819 |
| shsy5y | 30 | 30 | 30 |  |
| sjrh30 | 30 | 30 | 4.43692303 | 0.547425687 |
| moggccm | 30 | 30 | 7.01162052 |  |
| dld1 | 30 | 30 | 30 | 5.27448606 |
| sum44pe | 30 | 23.1260872 | 2.32703495 | 5.1745348 |
| c3a | 30 | 30 |  |  |
| pl45 | 30 | 30 | 12.2158737 | 0.022043495 |
| colo201 | 30 | 20.2380352 | 30 | 0.002049854 |
| kasumi3 | 30 | 2.79357338 | 30 | 0.672795594 |
